# Location-specific inhibition of Akt reveals regulation of mTORC1 activity in the nucleus

**DOI:** 10.1038/s41467-020-19937-w

**Published:** 2020-11-30

**Authors:** Xin Zhou, Yanghao Zhong, Olivia Molinar-Inglis, Maya T. Kunkel, Mingyuan Chen, Tengqian Sun, Jiao Zhang, John Y.-J. Shyy, JoAnn Trejo, Alexandra C. Newton, Jin Zhang

**Affiliations:** 1 Department of Pharmacology, University of California, San Diego, La Jolla, CA USA; 2 Biomedical Sciences Graduate Program, University of California, San Diego, La Jolla, CA USA; 3 Department of Bioengineering, University of California, San Diego, La Jolla, CA USA; 4 Division of Cardiology, Department of Medicine, University of California San Diego, La Jolla, CA USA; 5 Department of Chemistry & Biochemistry, University of California, San Diego, La Jolla, CA USA

**Keywords:** Growth factor signalling, Nucleus, Kinases

## Abstract

The mechanistic target of rapamycin complex 1 (mTORC1) integrates growth, nutrient and energy status cues to control cell growth and metabolism. While mTORC1 activation at the lysosome is well characterized, it is not clear how this complex is regulated at other subcellular locations. Here, we combine location-selective kinase inhibition, live-cell imaging and biochemical assays to probe the regulation of growth factor-induced mTORC1 activity in the nucleus. Using a nuclear targeted Akt Substrate-based Tandem Occupancy Peptide Sponge (Akt-STOPS) that we developed for specific inhibition of Akt, a critical upstream kinase, we show that growth factor-stimulated nuclear mTORC1 activity requires nuclear Akt activity. Further mechanistic dissection suggests that nuclear Akt activity mediates growth factor-induced nuclear translocation of Raptor, a regulatory scaffolding component in mTORC1, and localization of Raptor to the nucleus results in nuclear mTORC1 activity in the absence of growth factor stimulation. Taken together, these results reveal a mode of regulation of mTORC1 that is distinct from its lysosomal activation, which controls mTORC1 activity in the nuclear compartment.

## Abstract (web summary)

The role of mTORC1 at the lysosome is well established, but mTORC1 is known to be active in other cellular locations. Here, the authors develop Akt-STOPS to inhibit Akt specifically in the nucleus and identify a new regulatory mode for mTORC1 distinct in the nucleus.

## Introduction

The mechanistic target of rapamycin complex 1 (mTORC1), which contains the protein kinase mTOR, the key scaffolding protein Raptor, and several additional core components^1–3^, senses a wide range of intracellular and extracellular cues, including growth factors, amino acids, and cellular energy status^4^. When activated, mTORC1 incorporates these signals to coordinate a number of distinct cellular processes, such as cell growth and metabolism, by promoting the biosynthesis of macromolecules and energy production and suppressing the breakdown of proteins via inhibition of autophagy^4,5^. Activation of mTORC1 on the lysosomal surface is well documented in the literature^6–8^. Upon growth factor stimulation, protein kinase Akt phosphorylates the catalytic component of the tuberous sclerosis complex (TSC), dissociating it from the lysosomal surface and thereby relieving its negative regulation on Rheb, a direct and potent activator of mTORC1^7,9,10^. On the other hand, mTORC1 is also recruited and activated at the lysosome in Rag GTPase-dependent or independent manners in response to amino acid stimulation^11^. However, the regulation of mTORC1 within other subcellular compartments is less clear^12^. For instance, although mTOR and Raptor have been found in the nucleus by nuclear fractionation and immunofluorescence^13–22^, the presence of active and functional nuclear mTORC1 was debatable^13^. To examine nuclear mTORC1 activity, we developed a genetically encoded FRET-based biosensor, TORCAR, to visualize the activity dynamics of mTORC1 in living cells^23^. Briefly, TORCAR was constructed by sandwiching the mTORC1-specific substrate 4EBP1 between a pair of fluorescent proteins that can undergo FRET. Phosphorylation of TORCAR by endogenous mTORC1 induces a conformational change that leads to an increase in the emission ratio of cyan over yellow (C/Y)^23^. Using TORCAR targeted to the nucleus via nuclear localization signal (TORCAR-NLS), we showed that nuclear mTORC1 activity is stimulated by either growth factors or amino acid surrogates in serum- and amino acid-starved NIH3T3 cells^23^, providing initial evidence for active mTORC1 in the nucleus.

In this study, we focus on the regulation of nuclear mTORC1 activity. The serine/threonine kinase Akt is a critical component in the growth factor-stimulated mTORC1 signaling pathway. In the classical model, Akt critically regulates mTORC1 signaling at two different levels: Akt phosphorylates TSC2 within the aforementioned TSC complex and results in the activation of Rheb, a direct and potent activator of mTORC1; on the other hand, Akt phosphorylates PRAS40, an inhibitory component of mTORC1, leading to its dissociation from and activation of mTORC1^24,25^. Here we report a molecular tool, Akt-STOPS, that specifically suppresses Akt signaling at distinct subcellular localizations. Using Akt-STOPS, we discovered that nuclear mTORC1 activity is specifically dependent on the nuclear Akt activity. We further demonstrate that Akt facilitates nuclear translocation of Raptor, the regulatory component of mTORC1, which contributes to nuclear mTORC1 signaling. Thus, our findings reveal a non-canonical regulatory mechanism that specifically regulates a nuclear pool of mTORC1.

## Results

### Growth factor stimulates nuclear mTORC1 activity

**Fig. 1:**
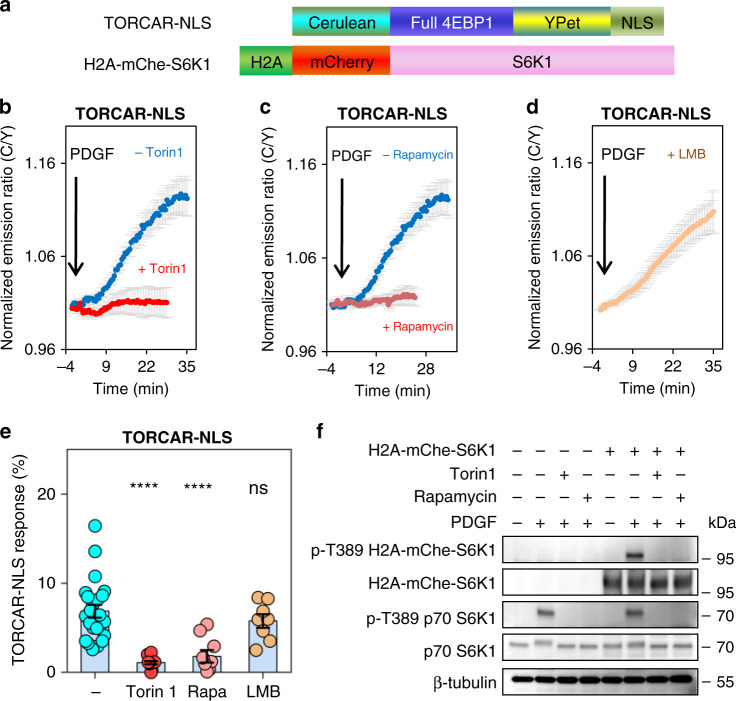
Growth factor stimulates mTORC1 activity in the nucleus. **a** Domain structures of nuclear TORCAR (TORCAR-NLS) and nuclear-localized S6K1 (H2A-mChe-S6K1). **b** Average time courses of normalized emission ratio (Cyan/Yellow) in double-starved NIH3T3 cells expressing nuclear-targeted TORCAR (TORCAR-NLS) stimulated with 50 ng/ml of PDGF without (blue trace, *n* = 23 cells) or with pretreatment with 1 µM of Torin1 for 10 min (red trace, *n* = 12 cells). Curves are representative of and pooled from four and three experiments, respectively. **c** Average time courses of normalized emission ratio (Cyan/Yellow) in double-starved NIH3T3 cells expressing nuclear-targeted TORCAR (TORCAR-NLS) stimulated with 50 ng/ml of PDGF without (blue trace, *n* = 23 cells) or with pretreatment with 50 µM of rapamycin for 10 min (red trace, *n* = 9 cells). Curves are representative of and pooled from four and five experiments, respectively. **d** Average time course of normalized emission ratio (Cyan/Yellow) in double-starved NIH3T3 cells expressing TORCAR-NLS pretreated with 100 nM of leptomycin B (LMB) for 30 min, followed by addition of 50 ng/ml of PDGF. Time course is representative of and pooled from three experiments. **e** Summary of responses of TORCAR-NLS in PDGF-treated double-starved NIH3T3 cells without pretreatment (*n* = 23), with Torin1 pretreatment (*n* = 12, ****, *p* = 1 × 10^−7^), with rapamycin pretreatment (*n* = 9, ****, *p* = 1 × 10^−5^), and with LMB pretreatment (*n* = 8, ns, not significant, *p* = 0.3). Error bar represents mean ± s.e.m. Ordinary one-way ANOVA followed by Dunnett’s multiple comparisons test. Source data are provided as a Source Data file. **f** Western blot analysis of double-starved NIH3T3 cells expressing H2A-mChe-S6K1 treated with Torin1 (1 µM) or rapamycin (50 µM) for 10 min prior to PDGF (50 ng/ml) stimulation for 30 min. Representative of three independent experiments. Full blots are shown in Supplementary Fig. 13.

Using a genetically encoded FRET-based mTORC1 activity reporter, TORCAR, we observed growth factor-induced mTORC1 activities across different subcellular locations, including the cytoplasm, lysosome, plasma membrane, and nucleus^23^. In particular, serum- and amino acid-starved (“double-starved”) NIH3T3 cells expressing nuclear-targeted TORCAR, TORCAR-NLS (Fig. 1a and Supplementary Fig. 1a), responded to the growth factor stimulation of PDGF with a 6.9 ± 0.7% increase in the emission ratio of cyan/yellow (C/Y) (Fig. 1b, blue trace, *n* = 23), showing a clear response despite the limited dynamic range of this first-generation mTORC1 activity reporter. Pretreatment with the mTOR kinase inhibitor Torin1 (Fig. 1b, red trace, 0.9 ± 0.1%, *p* < 0.0001, *n* = 12) or mTORC1 allosteric inhibitor rapamycin (Fig. 1c, red trace, 1.6 ± 0.8%, *p* < 0.0001, *n* = 9) inhibited the growth factor-induced response of TORCAR-NLS (Fig. 1e), suggesting the response is mTORC1 specific. Since NLS tagging does not prevent the reporter, TORCAR-NLS, from shuttling between the cytoplasm and the nucleus, we blocked the nuclear export pathway to examine whether the nuclear TORCAR response is reflecting mTORC1 activity exclusively within the nucleus. Treatment of 100 nM of leptomycin B (LMB), a specific inhibitor of the nuclear export receptor Chromosomal Maintenance 1 (CRM1), also known as Exportin 1, for 30 min effectively blocked the export pathway, as shown by nuclear accumulation of mCherry tagged with NES in NIH3T3 cells (Supplementary Fig. 1b). Under LMB treatment, double-starved NIH3T3 cells expressing TORCAR-NLS showed a response to PDGF with its amplitude on par with the responses of control cells (5.8 ± 0.8%, *n* = 8, ns) (Fig. 1d, e), suggesting that the nuclear TORCAR response is independent of CRM1-mediated nuclear export pathway and reports nuclear mTORC1 activity exclusively. To corroborate the observed nuclear mTORC1 activity, we anchored TORCAR to the nucleus by tagging histone 2A (H2A) to its N-terminus (H2A-TORCAR) (Supplementary Fig. 1c). Histone anchoring enhances the nuclear retention of tagged proteins, compared to tagging with NLS^26^, and therefore allows us to assess the response of TORCAR when it is more stringently confined to the nucleus. Like TORCAR-NLS, H2A-TORCAR is localized exclusively in the nucleus (Supplementary Fig. 1c) and showed a robust response of (purple trace, 4.0 ± 0.3%, *n* = 13, Supplementary Fig. 1d, e) to PDGF stimulation, which was sensitive to pretreatment with Torin1 (red trace, 0.4 ± 0.1%, *n* = 16, Supplementary Fig. 1d, f) or rapamycin (red trace, 0.4 ± 0.1%, *n* = 12, Supplementary Fig. 1e, f). Furthermore, in double-starved 3T3L1 adipocytes expressing H2A-TORCAR, stimulation with insulin induced a 4 ± 1% increase in C/Y ratio (*n* = 3, Supplementary Fig. 1g), suggesting nuclear mTORC1 activity can also be turned on by a metabolic regulator.

Next, we examined the phosphorylation of nuclear TORCAR. The addition of PDGF-induced phosphorylation of H2A-TORCAR (at the T37/46 sites within the 4EBP1 portion) by 6.1 ± 0.8 fold (Supplementary Figs. 1h and 14), whereas leptomycin B pretreatment failed to affect the level of H2A-TORCAR phosphorylation (92 ± 14% relative to no pretreatment, ns, Supplementary Figs. 1h and 14). As both TORCAR-NLS and H2A-TORCAR utilize full length 4EBP1 as a nuclear substrate surrogate, we further examined nuclear mTORC1 activity towards another established mTORC1-specific substrate, S6K1^27^. In cells expressing nuclear-targeted S6K1, H2A-mCherry-S6K1 (Fig. 1a, and Supplementary Fig. 1i), PDGF increased the level of phosphorylation of T389 S6K1 by 34 ± 4 fold (Fig. 1f and Supplementary Fig. 13), and S6K1 phosphorylation was sensitive to pretreatment of either Torin1 or rapamycin (Fig. 1f and Supplementary Fig. 13). The large increases in the phosphorylation of nuclear-confined mTORC1 substrates are indicative of robust nuclear mTORC1 activity. These results provide substantial evidence that mTORC1 is active in the nucleus and its activity can be stimulated by growth factor and insulin signaling pathways.

### Development of Akt-STOPS to perturb subcellular Akt signaling

**Fig. 2:**
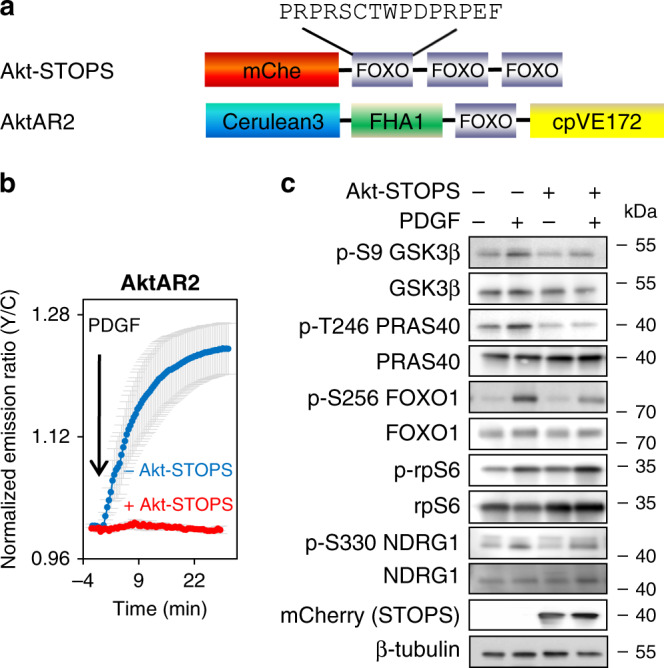
Development and characterization of Akt-STOPS. **a** Domain structures of Akt-STOPS and AktAR2. **b** Average time courses of normalized emission ratio (Yellow/Cyan) in serum-starved NIH3T3 cells expressing AktAR2 stimulated with 50 ng/ml of PDGF without (blue trace, 12 ± 1%, *n* = 41 cells) or with expression of Akt-STOPS (red trace, 0.02 ± 0.3%, *n* = 7 cells, ****, *p* = 4 × 10^−12^). Error bar represents mean ± s.e.m. Unpaired two-tailed *t* test with Welch’s correction. Curves are representative of and pooled from four and seven experiments, respectively. Source data are provided as a Source Data file. **c** Western blot analysis of serum-starved NIH3T3 cells with or without expressing Akt-STOPS treated with 50 ng/ml of PDGF for 30 min. Data were representative of three repeats. Full blots are shown in Supplementary Fig. 13.

The increase in mTORC1 activity in the nucleus may result from either recruitment of active mTORC1 into the nucleus or from activation of mTORC1 in the nucleus. To distinguish between these possibilities, we compared the kinetics of activity accumulation of mTORC1. We calculated the *t*_1/2_ of TORCAR responses as the time needed to reach 50% of the maximum amplitude, a metric used in previous studies^28–30^ (Supplementary Fig. 2a), and found mTORC1 activity in the nucleus, visualized by nuclear TORCARs, has an apparent *t*_1/2_ on par with the cytosolic accumulation of mTORC1 activity (*t*_1/2_ = 13.4 ± 0.9 min, *n* = 12 for TORCAR-NLS, 13 ± 1 min, *n* = 12 for H2A-TORCAR, and 12.4 ± 0.8 min, *n* = 25 for TORCAR; ns) (Supplementary Fig. 2a). We also performed curve fitting using an empirical sigmoidal function to describe the characteristics of the response curve, with a lag phase and a subsequent increase and found no evidence for faster kinetics for the untargeted TORCAR (Supplementary Fig. 2b–d). Overall, the comparison of kinetics of mTORC1 activity accumulation inside the nucleus and within cytosol does not allow us to rigorously determine if mTORC1 is activated in the nucleus in situ or translocated to the nucleus following activation. Since Akt critically regulates mTORC1 activity at multiple levels, including PRAS40 and TSC2, we reasoned that perturbing Akt signaling exclusively in the nucleus could allow us to examine the dependence of nuclear mTORC1 activity on nuclear Akt signaling. To develop a molecular tool to perturb specific pools of Akt activity using a substrate-based design^31^, we constructed Akt-STOPS (Akt Substrate-based Tandem Occupancy Peptide Sponge) by attaching three consecutive Akt substrate sequences derived from FoxO1 (Fig. 2a) to the C-terminus of a red fluorescent protein (mCherry). The design strategy was based on high affinity binding between Akt and its substrate sequence, which is typically stronger than a kinase and a pseudosubstrate^31,32^. The tandem repeats increase the avidity of the binding and allows the peptide to adopt multiple conformations to facilitate binding to Akt, thus buffering Akt activity. The red fluorescent protein serves both as a scaffold to display the flexible peptide and as a fluorescent tag to mark the location of inhibition. We first characterized Akt phosphorylation of the substrate peptide derived from FoxO1 which was utilized in the Akt-STOPS design. The in vitro kinase assay using the synthesized peptide (contains only one repeat of Akt substrate sequence) and purified Akt catalytic domain showed an apparent K_m_ for substrate of 126 ± 9 µM (black trace, Supplementary Fig. 3a), with undetectable phosphorylation of this substrate by protein kinase C βII (PKCβII) (red trace, Supplementary Fig. 3a).

We then tested the effects of untargeted Akt-STOPS on Akt activity in live cells by utilizing a FRET-based Akt activity reporter, AktAR2^23^. Like TORCAR, AktAR2 contains an Akt activity dependent molecular switch and generates a FRET change upon phosphorylation by Akt. While serum-starved NIH3T3 cells expressing AktAR2 showed an increase in Y/C emission ratio upon PDGF stimulation (12 ± 1%, *n* = 41), suggesting an increase in Akt activity, expression of untargeted Akt-STOPS abolished the PDGF-induced AktAR2 response (0.02 ± 0.3%, *n* = 7, ****, *p* < 0.0001, Fig. 2b). Expression of Akt-STOPS did not affect the cellular activity of protein kinase A (PKA) (21 ± 2%, *n* = 20), as shown by the unchanged response amplitude of A Protein Kinase Activity Reporter 4 (AKAR4)^33^ (20 ± 1%, *n* = 26), a FRET-based PKA activity reporter (Supplementary Fig. 3b). In addition, expressing mCherry alone (mChe) or mCherry-tagged scramble peptide (mChe-SP) (Supplementary Fig. 3c) did not reduce the AktAR2 response (Supplementary Fig. 3d). To further characterize the inhibitory effect of Akt-STOPS, we analyzed the growth factor-stimulated phosphorylation of Akt substrates (GSK3β, PRAS40, and FoxO1), an SGK substrate (NDRG1), and a p70S6K substrate (rpS6) in NIH3T3 cells transiently transfected with Akt-STOPS. Akt-STOPS expression reduced the phosphorylation of GSK3β, PRAS40, and FoxO1, but did not reduce the phosphorylation of NDRG1 and rpS6 (Fig. 2c, Supplementary Figs. 3e–i and 13). These results suggest that the cellular Akt activity can be specifically suppressed by Akt-STOPS.

**Fig. 3:**
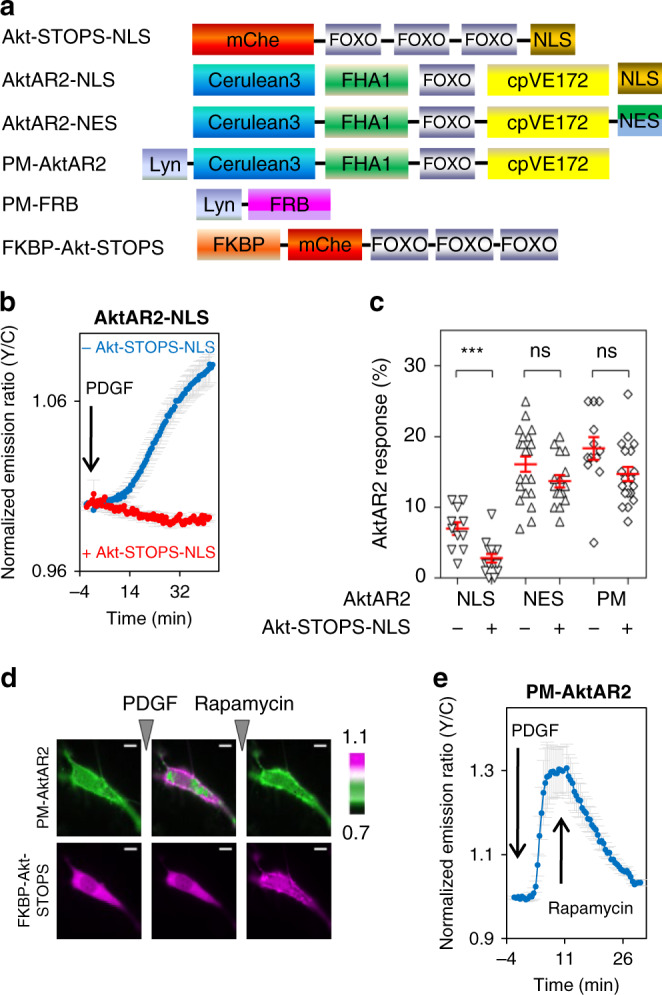
Akt-STOPS targeted to subcellular localizations perturbs local Akt signaling. **a** Domain structures of nuclear Akt-STOPS (Akt-STOPS-NLS), nuclear Akt Activity reporter 2 (AktAR2-NLS), cytosolic (AktAR2-NES), and plasma membrane AktAR2 (PM-AktAR2), as well as the Chemical Inducible Dimerization (CID) system using FKBP-rapamycin-FRB (PM-FRB and FKBP-Akt-STOPS). **b** Average time course of normalized emission ratio (Yellow/Cyan) in serum-starved NIH3T3 cells expressing AktAR2-NLS stimulated with 50 ng/ml of PDGF without (blue trace, *n* = 11 cells) or with expression of Akt-STOPS-NLS (red trace, *n* = 15 cells). Error bar represents mean ± s.e.m. Curves are representative of and pooled from four and seven experiments, respectively. **c** Summary of responses of PDGF-treated serum-starved NIH3T3 cells expressing subcellular targeted AktAR2 with or without co-expression of Akt-STOPS-NLS. Error bar represents mean ± s.e.m. *P*-value was determined by unpaired two-tailed Student’s *t* test. The responses of AktAR2-NLS without and with Akt-STOPS-NLS are 7.0 ± 0.8% (*n* = 11 cells from four experiments) and 2.7 ± 0.6% (*n* = 15 cells from seven experiments), respectively, ***, *p* = 2 × 10^−4^; the responses of AktAR2-NES without and with Akt-STOPS-NLS are 16 ± 1% (*n* = 21) and 13.6 ± 0.8% (*n* = 16), respectively, ns, *p* = 0.1; the responses of PM-AktAR2 without and with Akt-STOPS-NLS are 18 ± 1% (*n* = 17) and 15 ± 1% (*n* = 16), respectively, ns, not significant, *p* = 0.1. Source data are provided as a Source Data file. **d** Images of serum-starved NIH3T3 cells expressing the CID system (as shown in **a**). Representative of three independent experiments. Upper panel: pseudocolor images showing the PM-AktAR2 response. Warmer colors indicating higher normalized ratios of Yellow/Cyan for PM-AktAR2. Lower panel: dynamics of FKBP-Akt-STOPS. Arrows indicating the addition of PDGF or rapamycin. Scale bar = 10 µm. **e** Average time course of normalized emission ratio (Yellow/Cyan) in serum-starved NIH3T3 cells expressing PM-AktAR2, PM-FRB, and FKBP-Akt-STOPS showed a response to PDGF stimulation (50 ng/ml) that was reversed by the addition of rapamycin (100 nM), *n* = 7. Time course is representative of and pooled from three experiments.

We then tested whether nuclear-targeted Akt-STOPS can be used to specifically perturb nuclear Akt activity. Expression of Akt-STOPS-NLS (Fig. 3a) decreased the PDGF-induced response of nuclear Akt activity reporter (AktAR2-NLS) (***, *p* < 0.001, Fig. 3b, c). Importantly, the nuclear-targeted Akt-STOPS did not significantly change cytosolic or plasma membrane Akt activity, as shown by the response of cytosolic Akt activity reporter (AktAR2-NES) or the plasma membrane Akt activity reporter (PM-AktAR2) (ns, Fig. 3c and Supplementary Fig. 4a). Expression of Akt-STOPS-NLS also decreased the PDGF-induced phosphorylation of PRAS40 in serum-starved NIH3T3 cells (Supplementary Fig. 4b, c). These results suggested that Akt-STOPS-NLS selectively suppressed nuclear Akt activity.

We also developed plasma membrane-targeted Akt-STOPS (PM-Akt-STOPS, Supplementary Fig. 5a) by attaching a lipid modified domain from Lyn to the N-terminus to Akt-STOPS. PM-Akt-STOPS dampened the PDGF-induced responses of plasma membrane Akt activity (****, *p* < 0.0001) with little effect on the Akt activity in the cytosol (ns, *p* = 0.06) (Supplementary Fig. 5b–d). Interestingly, nuclear Akt activity was also decreased by expression of plasma membrane Akt-STOPS (***, *p* < 0.001) (Supplementary Fig. 5b–d), suggesting that nuclear Akt activity may have some dependence on plasma membrane Akt activity. To further develop an approach that allows temporal control on the location-specific inhibition of Akt activity, we employed the Chemical Inducible Dimerization (CID) system based on rapamycin-induced heterodimerization of FK506-binding proteins (FKBP) and FKBP12-rapamycin Binding (FRB) domain. In these studies, we transiently transfected NIH3T3 cells with FKBP-Akt-STOPS together with a plasma membrane-targeted FRB (PM-FRB) (Fig. 3a) and monitored Akt activity at the plasma membrane using PM-AktAR2. Upon addition of the dimerizer rapamycin, a ternary complex of FKBP-rapamycin-FRB is formed, resulting in the recruitment of Akt-STOPS to the plasma membrane (Fig. 3d). As shown in Fig. 3d, e, addition of rapamycin following PDGF, led to the rapid reversal of PDGF-stimulated PM-AktAR2 response, suggesting acute recruitment of Akt-STOPS to the plasma membrane inhibits the Akt activity in situ. Similarly, the addition of rapalog^34,35^, which avoids the potential interference via inhibition of mTORC1, also rapidly reversed the PDGF-induced PM-AktAR2 responses (Supplementary Fig. 5e), suggesting Akt-STOPS is a powerful molecular tool for spatiotemporal suppression of Akt activity in living cells.

### Nuclear Akt activity is required for growth factor-induced nuclear mTORC1 activity

**Fig. 4:**
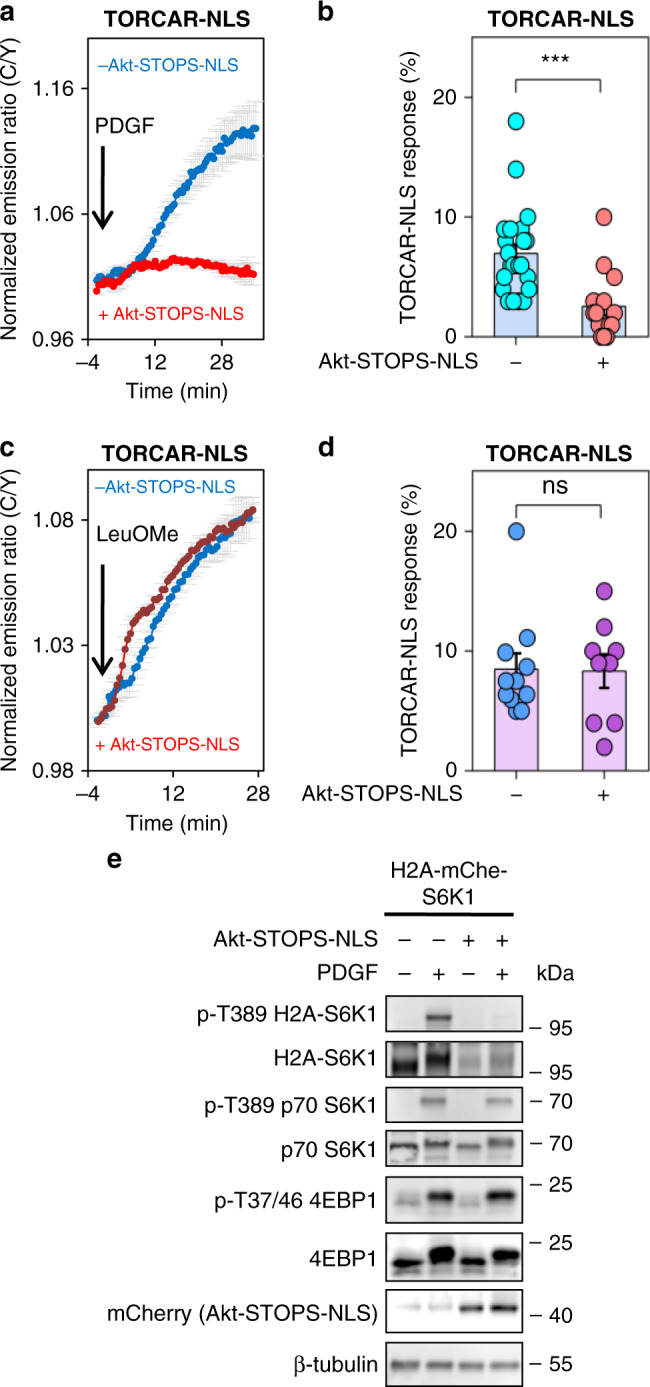
Nuclear Akt activity is required for growth factor-induced nuclear mTORC1 activity. **a** Average time courses of normalized emission ratio (Cyan/Yellow) in double-starved NIH3T3 cells expressing TORCAR-NLS stimulated with 50 ng/ml of PDGF without (blue trace, *n* = 23 cells from four experiments) or with expression of Akt-STOPS-NLS (red trace, *n* = 14 cells from three experiments). Curves are representative of and pooled from four and three experiments, respectively. **b** Responses of TORCAR-NLS in PDGF-treated double-starved NIH3T3 cells without (*n* = 23) or with expression of Akt-STOPS-NLS (*n* = 14). Error bar represents mean ± s.e.m. *P*-value was determined by unpaired two-tailed Student’s *t* test. ***, *p* = 4 × 10^−4^. Source data are provided as a Source Data file. **c** Average time courses of normalized emission ratio (Cyan/Yellow) in double-starved NIH3T3 cells expressing TORCAR-NLS stimulated with 7.5 mM leucine O-methyl ester (LeuOMe) without (blue trace, *n* = 11 cells) or with expression of Akt-STOPS-NLS (red trace, *n* = 9 cells). Curves are representative of and pooled from five and three experiments, respectively. **d** Responses of TORCAR-NLS in LeuOMe-treated double-starved NIH3T3 cells without (*n* = 11 cells from five experiments) or with expression of Akt-STOPS-NLS (*n* = 9 cells from three experiments). Error bar represents mean ± s.e.m. Unpaired two-tailed Student’s *t* test. ns, not significant, *p* = 1.0. Source data are provided as a Source Data file. **e** Western blot analysis of double-starved NIH3T3 cells expressing H2A-mChe-S6K1 treated with PDGF (50 ng/ml) for 30 min in the absence or presence of STOPS-NLS expression. Representative of three independent experiments. Full blots are shown in Supplementary Fig. 13.

The location-selective inhibition by Akt-STOPS-NLS permitted the analysis of whether the nuclear mTORC1 activity is primarily dependent on the nuclear Akt activity. Intriguingly, in double-starved NIH3T3 cells expressing nuclear-targeted mTORC1 activity reporter (TORCAR-NLS), the presence of Akt-STOPS-NLS led to a 63 ± 11% inhibition of PDGF-stimulated TORCAR-NLS response (red trace, 2.7 ± 0.7%, *n* = 14), compared with control cells (blue trace, 6.9 ± 0.7%, *n* = 23, ***, *p* < 0.001) (Fig. 4a, b). In contrast, expression of a nuclear-targeted scramble peptide failed to reduce nuclear TORCAR response (green trace, 9 ± 1%, *n* = 10, ns, Supplementary Fig. 6a–c). In addition, the nuclear TORCAR response to PDGF is significantly reduced by pretreatment with Akt inhibitors (GDC-0068 and MK-2206), but not by pretreatment with H89 (PKA inhibitor), Gö6983 (PKC inhibitor), PF-4708671 (p70 S6K inhibitor), LJI308 (p90 RSK inhibitor), or GSK650394 (SGK inhibitor) (Supplementary Fig. 6d, e), suggesting that growth factor-induced nuclear mTORC1 activity specifically depends on Akt activity. As an additional control, nuclear mTORC1 activity stimulated by an amino acid surrogate leucine O-methyl ester (LeuOMe) was examined and not affected by Akt-STOPS-NLS (8 ± 1% vs 8 ± 1%, *n* = 11 and 9, ns, Fig. 4c, d), indicating that the amino acid surrogate-induced nuclear mTORC1 activity is independent of nuclear Akt, as expected. Consistent with the TORCAR data, western blot analysis showed that expression of nuclear Akt-STOPS (Akt-STOPS-NLS) abolished the phosphorylation of H2A-mChe-S6K1 without affecting the phosphorylation of endogenous S6K1 and 4EBP1, which are primarily localized outside the nucleus (Fig. 4e and see Supplementary Fig. 13 for longer exposure). Together, these data suggest that nuclear Akt activity is critical for the growth factor-stimulated nuclear mTORC1 activity.

### Raptor forms a complex with mTOR in the nucleus

**Fig. 5:**
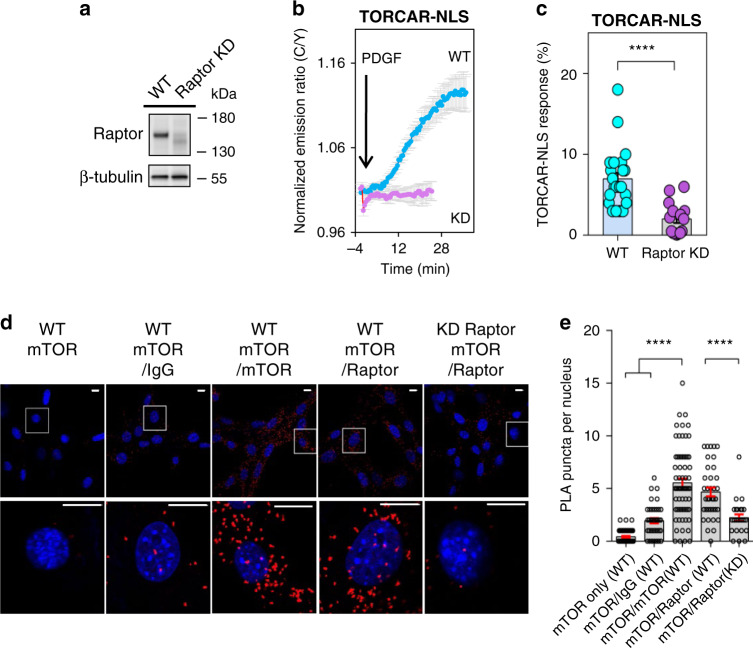
Raptor is required for nuclear TORCAR response and forms a complex with mTOR in the nucleus. **a** shRNA-mediated knockdown of Raptor in NIH3T3 cells. Western blot analysis showed reduced level in Raptor KD cells. Representative of three independent experiments. Full blots are shown in Supplementary Fig. 13. **b** Average time courses of normalized emission ratio (Cyan/Yellow) in double-starved wild-type (WT, blue trace, *n* = 23 cells) or Raptor knockdown (KD, purple trace, *n* = 15 cells) NIH3T3 cells expressing nuclear-targeted TORCAR (TORCAR-NLS) stimulated with 50 ng/ml of PDGF. Time course is representative of and pooled from four and four experiments, respectively. **c** Responses of TORCAR-NLS to PDGF in WT (*n* = 23 cells from four experiments) and Raptor KD NIH3T3 cells (*n* = 15 cells from four experiments). Error bar represents mean ± s.e.m. Unpaired two-tailed Student’s *t* test with Welch’s correction. ****, *p* = 5 × 10^−6^. Source data are provided as a Source Data file. **d** Representative confocal images of in situ proximity ligation assay (PLA) between mTOR and Raptor (red) in wild type NIH3T3 cells (Column 1, 2, 3, 4) or Raptor knockdown NIH3T3 cells (Column 5). Column 1, mouse anti-mTOR only; column 2, mouse anti-mTOR and IgG; column 3, rabbit anti-mTOR antibody and mouse anti-mTOR antibody; column 4 and 5, mouse anti-mTOR and rabbit anti-Raptor antibodies. The middle slices of confocal z-stacks were shown to observe the PLA puncta (red) in the nucleus (blue). Data are representative of two independent experiments. Scale bar = 10 µm. **e** Quantification of the amount of PLA puncta per nucleus. *n* = 50, 37, 69, 37, and 26 cells. Error bar represents mean ± s.e.m. Ordinary one-way ANOVA followed by Tukey’s multiple comparisons test. mTOR only (WT) vs. mTOR/mTOR (WT), ****, *p* = 5 × 10^−41^; mTOR/IgG(WT) vs. mTOR/mTOR (WT), ****, *p* = 7 × 10^−22^; mTOR/Raptor (KD) vs. mTOR/Raptor (WT), ****, *p* = 2 × 10^−8^. Source data are provided as a Source Data file.

Raptor is the critical and defining component for mTORC1. Within the mTORC1 complex, Raptor binds to the TOR signaling (TOS) motif on mTORC1 substrates to determine substrate specificity and aids the catalysis by mTOR^27,36^. To examine whether Raptor is required for nuclear TORCAR response, we employed shRNA-mediated knockdown to decrease the protein level of Raptor significantly in NIH3T3 cells (Fig. 5a and Supplementary Fig. 13). In these Raptor knockdown NIH3T3 cells, PDGF-stimulated TORCAR-NLS response was significantly decreased (2.0 ± 0.5%, *n* = 15, ****, *p* < 0.0001, Fig. 5b, c), suggesting Raptor is essential for nuclear mTORC1 activity.

We then examined whether mTOR and Raptor form a complex in the nucleus by proximity ligation assay (PLA). In these experiments, complexes containing mTOR and Raptor were detected in intact, fixed cells. As shown in Fig. 5d, the PLA signal was observed in both the cytoplasm and nucleus. On average, 4.7 ± 0.4 puncta per nucleus (*n* = 37 cells) were observed for the measurements of mTOR and Raptor interaction in wild-type NIH3T3 cells, which is significantly higher than the background signals observed with either mTOR antibody alone (0.4 ± 0.1 dots per nucleus, *n* = 50 cells, ****, *p* < 0.0001) or mTOR antibody with normal IgG (2.0 ± 0.3 dots per nucleus, *n* = 37 cells, ****, *p* < 0.0001). As a control, PLA signals decreased to 2.2 ± 0.3 dots per nucleus in Raptor knockdown NIH3T3 cells (*n* = 26 cells, ****, *p* < 0.0001) (Fig. 5e). These data suggest that mTOR and Raptor form a complex in the nucleus.

### Raptor translocates into the nucleus upon PDGF stimulation

**Fig. 6:**
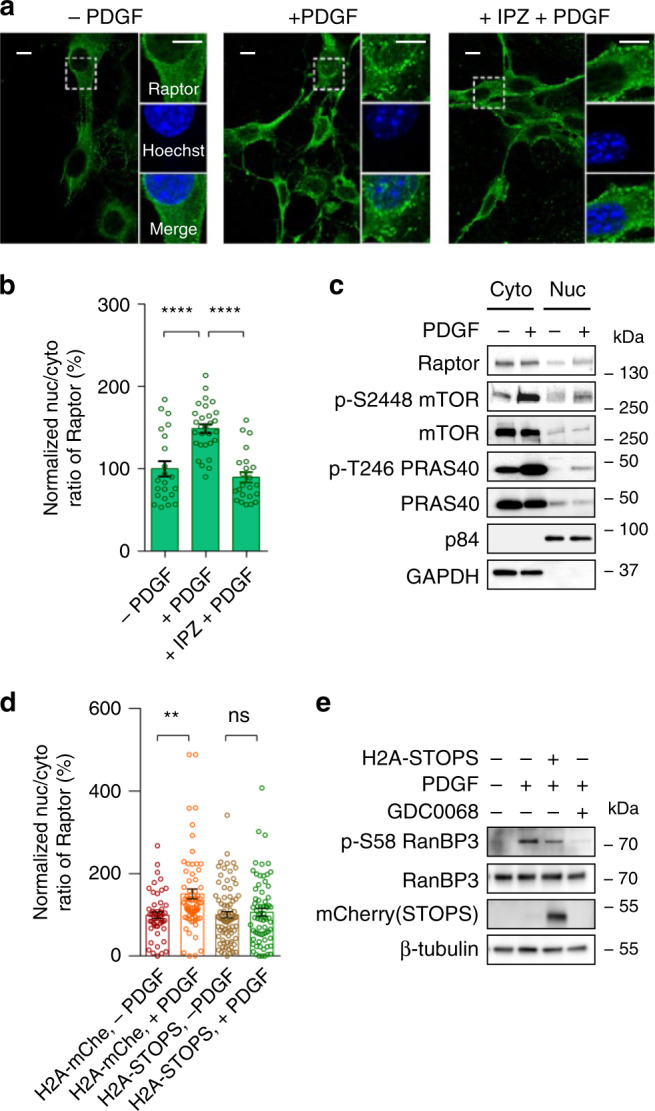
Raptor translocation into the nucleus upon growth factor stimulation is dependent on nuclear Akt activity. **a** Confocal images of immunostaining of endogenous Raptor in double-starved NIH3T3 cells (−PDGF), 30 min following PDGF stimulation (+PDGF), and treatment with 100 µM importazole (IPZ) for 1 hr followed by PDGF stimulation for 30 min (+IPZ + PDGF). The middle slices of confocal z-stacks were shown to observe the Raptor staining in the nucleus (Green). Scale bar = 10 µm. *n* = 3 experiments. **b** Quantification of mean nuclear intensity/mean cytosol intensity ratio (Nuc/Cyto ratio) of Raptor immunostaining per cell. *n* = 22, 29, 23 cells from three experiments. Error bar represents mean ± s.e.m. One-way ANOVA followed by Tukey’s tests. −PDGF vs. +PDGF, ****, *p* = 6 × 10^−10^; +PDGF vs. +IPZ + PDGF, ****, *p* = 6 × 10^−13^. Source data are provided as a Source Data file. **c** Western blot analysis of cytoplasmic and nuclear fractions from double-starved NIH3T3 cells treated without or with PDGF (50 ng/ml) for 30 min. P84 and GAPDH were used as nuclear and cytoplasmic markers, respectively. Data are representative of three experiments. Full blots are shown in Supplementary Fig. 13. **d** Summary of changes in Nuc/Cyto ratio of Raptor immunostaining in double-starved NIH3T3 cells expressing mCherry (H2A-mChe) or Akt-STOPS in the nucleus (H2A-STOPS) stimulated without or with PDGF (50 ng/ml) for 30 min. *n* = 49, 78, 82, and 71 cells from three experiments. Error bar represents mean ± s.e.m. One-way ANOVA followed by Tukey’s tests. H2A-mChe, −PDGF vs. H2A-mChe, +PDGF, **, *p* = 2 × 10^−6^; H2A-STOPS, −PDGF vs. H2A-STOPS, +PDGF, ns, not significant, *p* = 0.5. Source data are provided as a Source Data file. **e**. Western blot analysis of phosphorylation of RanBP3 in double-starved NIH3T3 cells with different treatment indicated. Lane 1, no treatment; Lane 2, PDGF (50 ng/ml) treatment for 30 min; Lane 3, cells expressing H2A-STOPS were treated with PDGF (50 ng/ml) for 30 min; Lane 4, pretreatment with GDC-0068 (1 µM) for 10 min followed by PDGF (50 ng/ml) for 30 min. Representative of three independent experiments. Full images of blots are shown in Supplementary Fig. 13.

Using an antibody validated for Raptor immunofluorescence (Supplementary Fig. 7a, b), we examined Raptor localization before and after PDGF stimulation. As shown in Fig. 6a, b, PDGF treatment of double-starved NIH3T3 cells resulted in a reproducible increase in nuclear accumulation of endogenous Raptor, with a 49 ± 6% increase in the nuclear/cytosol (Nuc/Cyto) ratio of the immunofluorescence signal of Raptor. To validate Raptor’s nuclear localization after PDGF treatment, we utilized whole lysate nuclear extraction to assess the levels of Raptor in control and PDGF-treated NIH3T3 cells. Quantifying Raptor levels normalized to a fraction marker (GAPDH and p84 for cytoplasmic and nuclear fractions, respectively), we observed elevated and decreased Raptor protein levels in nuclear and cytosolic fractions, respectively, following growth factor stimulation (Fig. 6c, Supplementary Figs. 8, 13 and 14), although our protocol is not suited for comparing protein abundance between cytosolic and nuclear fractions (see “Methods”). PDGF stimulation also increased the phosphorylation of mTOR at S2448 and PRAS40 at T426 but does not change the total levels of Raptor in whole cell lysates (Fig. 6c, Supplementary Figs. 8b, 13, 14). Phosphorylated mTOR at S2448 is a marker for active mTORC1^37^, and the observed increased phosphorylation is consistent with increased nuclear mTORC1 activity.

To validate PDGF-induced Raptor nuclear translocation, the nuclear import pathway was blocked using a small molecule inhibitor importazole (IPZ), which inhibits importin β^38^. Treatment of NIH3T3 cells with 100 µM of importazole for 1 h efficiently blocked accumulation of mCherry-NLS in the nucleus (Supplementary Fig. 7c), suggesting this treatment is sufficient to inhibit the nuclear import of protein tagged with an NLS. As shown in Fig. 6a, b, pretreatment with IPZ abolished the PDGF-induced nuclear translocation of Raptor in serum and amino acid-starved NIH3T3 cells, suggesting that Raptor nuclear translocation is mediated by Ran/importin pathway.

We further examined whether Raptor nuclear translocation specifically depends on the nuclear Akt activity. Akt inhibition by the small molecule inhibitor MK2206 decreased the PDGF-induced nuclear Raptor translocation (Supplementary Fig. 7d, e). Furthermore, as shown in Fig. 6d, the PDGF-induced increase in the nuc/cyto ratio of Raptor immunofluorescence signal was blocked in cells expressing nuclear-targeted Akt-STOPS (H2A-Akt-STOPS), but not in cells expressing nuclear-targeted mCherry (H2A-mChe), indicating that this process is dependent on nuclear Akt activity.

Akt is known to phosphorylate Ser58 of the Ran GTPase binding protein 3 (RanBP3) to facilitate the Ran GTPase-mediated nuclear import^39^. Given the nuclear localization of RanBP3 (Supplementary Fig. 7f), we hypothesized that Akt-mediated RanBP3 phosphorylation primarily occurs in the nucleus and that nuclear Akt activity is important for regulating RanBP3. To test this idea, NIH3T3 cells were transiently transfected with nuclear-targeted Akt-STOPS (Akt-STOPS-NLS), double-starved and stimulated with PDGF, and the phosphorylation of RanBP3 at Ser58 was examined. Although transfection efficiency limited the effect of Akt-STOPS-NLS across the entire cell population, Akt-STOPS-NLS led to a 49 ± 6% decrease in RanBP3 phosphorylation (Fig. 6e). These data suggest that nuclear Akt activity mediates phosphorylation of RanBP3, which may contribute to nuclear translocation of Raptor.

### Nuclear-localized Raptor potentiates nuclear TORCAR response and results in nuclear mTORC1 activity in the absence of growth factor stimulation

**Fig. 7:**
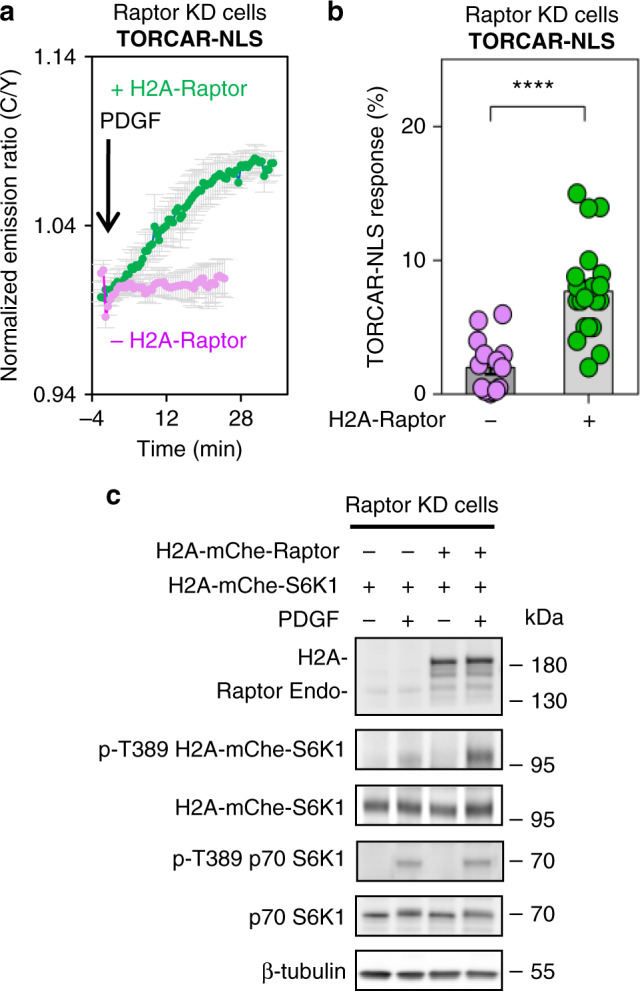
Expressing nuclear-targeted Raptor rescues nuclear mTORC1 activity. **a** Average time courses of normalized emission ratio (Cyan/Yellow) in double-starved Raptor knockdown NIH3T3 cells expressing nuclear-targeted TORCAR (TORCAR-NLS) stimulated with 50 ng/ml of PDGF without (magenta trace, *n* = 15 cells) or with expression of H2A-Raptor (green trace, *n* = 23 cells). Curves are representative of and pooled from four and three experiments, respectively. **b** Responses of TORCAR-NLS in PDGF-treated double-starved Raptor knockdown NIH3T3 cells without (*n* = 15 cells from four experiments) or with H2A-Raptor expression (*n* = 23 cells from three experiments). Error bar represents mean ± s.e.m. *P*-value was determined by unpaired two-tailed Student’s *t* test with Welch’s correction. ****, *p* = 1 × 10^−6^. Source data are provided as a Source Data file. **c** Western blot analysis of double-starved Raptor knockdown NIH3T3 cells expressing H2A-mChe-S6K1 with or without expression of H2A-Raptor treated with PDGF (50 ng/ml) for 30 min. Representative of three independent experiments. Full blots are shown in Supplementary Fig. 13.

Our data suggest that growth factor-induced nuclear translocation of Raptor may be important for the increase in nuclear mTORC1 activity. We next examined the effect of localizing Raptor to the nucleus. We first utilized the Raptor shRNA-knockdown NIH3T3 cells and expressed a nuclear-localized Raptor (H2A-Raptor) and examined its effect on nuclear TORCAR response. Expression of H2A-Raptor rescued the PDGF-induced response of TORCAR-NLS in double-starved Raptor knockdown cells (2.0 ± 0.5% vs 7.7 ± 0.8%, *n* = 15 and 23, ***, *p* < 0.0005, Fig. 7a, b). In contrast, overexpression of nuclear Raptor had no effect on cytosolic TORCAR (TORCAR-NES, Supplementary Fig. 9a) in Raptor knockdown NIH3T3 cells (0.9 ± 0.2% vs 1.9 ± 0.5%, *n* = 11 and 7, ns, Supplementary Fig. 9b, c), while cytosolic TORCAR is clearly functional in wild-type NIH3T3 cells (Supplementary Fig. 9d, e). These data are consistent with the 2.8 ± 0.2 fold increase in PDGF-induced phosphorylation of nuclear-localized H2A-S6K1 in the presence of nuclear Raptor (H2A-Raptor) in Raptor knockdown cells compared to that in the absence of nuclear Raptor (Fig. 7c). These results suggest that nuclear localization of Raptor is necessary for rescuing nuclear mTORC1 activity in Raptor knockdown cells.

**Fig. 8:**
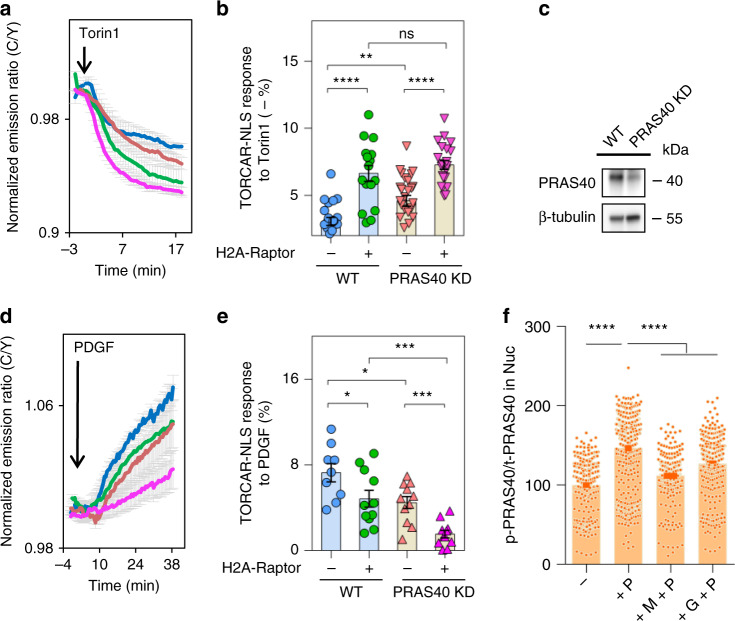
Nuclear TORCAR response is influenced by nuclear-localized Raptor and PRAS40. **a** Torin1-induced responses in serum-starved NIH3T3 cells or PRAS40 knockdown (KD) NIH3T3 cells expressing nuclear-targeted TORCAR (TORCAR-NLS) without (Blue, *n* = 22 WT cells, seven experiments; Red, *n* = 26 KD cells, five experiments) or with H2A-Raptor co-expression (Green, *n* = 16 WT cells, five experiments; Pink, *n* = 22 KD cells, seven experiments). **b** Basal activities indicated by Torin1-induced decreases (−%) in **a**. Error bar represents mean ± s.e.m. Unpaired two-tailed Student’s *t* test. WT vs. WT + H2A-Raptor, ****, *p* = 2 × 10^−6^ (with Welch’s correction); KD vs. KD + H2A-Raptor, ****, *p* = 8 × 10^−6^; WT vs. KD, **, *p* = 5 × 10^−3^; WT + H2A-Raptor vs. KD + H2A-Raptor, ns, not significant, *p* = 0.3. Source data are provided as a Source Data file. **c** shRNA-mediated knockdown of PRAS40 in NIH3T3 cells. Representative of three independent experiments. Full blots are shown in Supplementary Fig. 13. **d** PDGF-induced responses in serum-starved WT cells or PRAS40 KD cells expressing TORCAR-NLS without (Blue, *n* = 9 WT cells, three experiments; Red, *n* = 11 KD cells, five experiments) or with H2A-Raptor co-expression (Green, *n* = 11 WT cells, three experiments; Pink, *n* = 11 KD cells, three experiments). **e** PDGF-induced responses in **c**. Error bar represents mean ± s.e.m. Unpaired two-tailed Student’s *t* test. WT vs. WT + H2A-Raptor, *, *p* = 5 × 10^−3^; KD vs. KD + H2A-Raptor, ***, *p* = 3 × 10^−4^; WT vs. KD, *, *p* = 1 × 10^−2^; WT + H2A-Raptor vs. KD + H2A-Raptor, **, *p* = 2 × 10^−3^ (with Welch’s correction). **f** Quantification of immunostaining of mean nuclear p-T246 PRAS40 intensity/total PRAS40 intensity ratio (p-PRAS40/PRAS40 in Nuc) per cell. Double-starved NIH3T3 cells without treatment (−), 30 min following PDGF stimulation (+P), and pretreatment with 1 µM MK-2206 (+M + P) or GDC-0068 (+G + P) for 10 min followed by 30 min treatment with PDGF were co-stained with p-T246 and total PRAS40 antibodies. *n* = 415, 499, 280, 366 cells. Data are pooled from three experiments. Error bar represents mean ± s.e.m. One-way ANOVA with Tukey’s test. −P vs. +P, ****, *p* = 3 × 10^−130^; +P vs. +M + P, ****, *p* = 3 × 10^−65^; +P vs. +G + P, ****, *p* = 3 × 10^−26^. Source data are provided as a Source Data file.

We next examined how nuclear-localized Raptor affects the basal and stimulated nuclear mTORC1 activities in wild-type cells. To directly evaluate basal nuclear mTORC1 activity, we treated resting/unstimulated TORCAR-NLS-expressing cells with the mTOR inhibitor, Torin1, and quantified the resulting decrease in the TORCAR-NLS cyan-over-yellow emission ratio. This technique is widely used to characterize basal pathway activation for other kinases such as PKA^33^ and PKC^40–42^. As shown in Fig. 8a, b, Torin1 induced a larger decrease in TORCAR-NLS C/Y ratio in cells expressing nuclear Raptor than in cells without expressing nuclear Raptor (6.6 ± 0.6% vs 3.1 ± 0.3%, *n* = 16 and 22, respectively, ****, *p* < 0.0001), and this decrease is further enhanced (7.3 ± 0.3% and 4.6 ± 0.4%, *n* = 22 and 26 for cells with and without expressing H2A-Raptor, respectively, ****, *p* < 0.0001) by shRNA knockdown of PRAS40 (Fig. 8c and Supplementary Fig. 13), a negative mTORC1 regulator and Akt substrate^43,44^. On the other hand, tethering Raptor to the nucleus resulted in reduced amplitude of PDGF-induced nuclear TORCAR response (7.3 ± 0.8 vs 4.8 ± 0.8, *n* = 9 and 11, *, *p* < 0.05), which is further reduced in PRAS40 knockdown cells (4.5 ± 0.6% vs. 1.5 ± 0.4, *n* = 11 and 11, ***, *p* < 0.0005, Fig. 8d, e). Taken together, these results suggest the basal nuclear mTORC1 activity in wild-type cells is low, and localizing additional Raptor to the nucleus increases the basal activity of nuclear mTORC1, suggesting that localization of Raptor to the nucleus itself can result in nuclear mTORC1 activity in the absence of growth factor stimulation. Furthermore, these results suggest that PRAS40, which is phosphorylated in the nucleus by Akt upon PDGF stimulation (Figs. 6c, 8f), also contributes to the PDGF-induced increase in nuclear mTORC1 activity.

### Nuclear Akt-STOPS suppresses pol III transcription

mTORC1 has been shown to play an important role in regulating the synthesis of tRNA and 5S rRNA by RNA polymerase (pol) III^45,46^. mTOR was found at tRNA and 5S rRNA genes in the nucleus^45,46^, where mTOR associates with TFIIIC, a transcription factor that binds to pol III promoters. Maf1, a repressor that binds and inhibits pol III, was shown to be phosphorylated in an mTOR-dependent manner at serine 75, a site that is important for its function as a transcriptional repressor. These results led to the model proposing that mTORC1 phosphorylates Maf1 at the promoters, relieving its repression on pol III-dependent transcription of tRNA and 5S rRNA (Supplementary Fig. 10a)^15^. To test whether nuclear mTORC1 activity regulates pol III-dependent transcription, we examined the effect of nuclear Akt-STOPS on pol III-dependent transcription of tRNAs. We compared the expression of pre-tRNA^Leu^ and pre-tRNA^Tyr^ in NIH3T3 cells expressing Akt-STOPS-NLS. The presence of Akt-STOPS-NLS down-regulated pol III transcripts tRNA^Leu^ and tRNA^Tyr^ by 2.3- and 1.8-fold, respectively, in agreement with the inhibition of transcription by rapamycin treatment (Supplementary Fig. 10b). This data suggests Akt-STOPS-NLS negatively regulates the pol III transcription apparatus, consistent with a positive role of nuclear mTORC1 in regulating pol III-mediated transcription.

## Discussion

The kinase complex mTORC1 serves as a central signaling node to transduce diverse signal inputs that control cell growth and metabolism. The intricate spatial regulation of mTORC1 allows downstream signaling to be specific and efficient. As the best characterized site for mTORC1 function, the lysosomal surface has been suggested to be a cellular location where growth factors and nutrient signals converge. An intriguing question is whether mTORC1 activity exists at other subcellular sites to exert broader functional control of downstream effectors. In this study, we used a genetically encoded FRET-based biosensor, in combination with an exogenous nuclear-targeted mTORC1 substrate S6K1, to provide clear evidence that mTORC1 activity is present in the nucleus where it can be stimulated by growth factors and insulin.

Given the critical regulation of mTORC1 by Akt downstream of growth factor signaling, we developed Akt-STOPS to perturb the Akt signaling in a location-specific manner. Previously, a location-specific Akt inhibitor, PKBi, was developed based on kinase trapping by a designed pseudosubstrate of Akt^32^. Using a similar approach, but taking advantage of strong inhibitory effects by a kinase substrate compared to a pseudosubstrate, we developed AMPK inhibitor peptide (AIP) using the consensus substrate peptide for AMPK^31^. Prompted by these studies, we designed Akt-STOPS to buffer Akt activity towards endogenous substrates. These genetically encodable peptide inhibitors (GEPIs), either in pseudosubstrate- or substrate-competitive manner, usually exhibit IC_50_ in high µM range^31,47^ in vitro, in comparison with small molecule inhibitors with IC_50_ in low µM or even nM range, to inhibit the activity of kinases-of-interest. However, these genetically encoded peptide inhibitors can achieve effective inhibition in cells and offer a unique advantage as they can be targeted to specific subcellular locations, membrane microdomains, or multi-protein complexes^31^, allowing the perturbation of the cellular enzyme activities with high spatial precision.

In this study, by using Akt-STOPS, we found nuclear mTORC1 activity is dependent on nuclear Akt activity upon growth factor stimulation but not amino acid surrogate stimulation. Our findings further suggest a model where nuclear Akt facilitates the nuclear translocation of Raptor (Supplementary Fig. 12). Raptor is a critical and defining component of the mTORC1 complex and binds several mTORC1 substrates to the TOR signaling (TOS) motif^36,48^. In response to amino acids, Raptor binds to Rag GTPases to localize mTOR to the lysosomal surface^49–51^. Our data showed that localizing exogenous Raptor to the nucleus results in nuclear mTORC1 activity in the absence of growth factor stimulation. An attractive model would be that in the presence of growth factor, Raptor translocates into the nucleus and allows mTORC1 to assemble in the nucleus, leading to an increase in nuclear mTORC1 activity. Interestingly, expression of nuclear Raptor slightly increased the nuclear distribution of mTOR upon growth factor stimulation (Supplementary Fig. 11), whereas no significant changes in mTOR nuclear localization was observed in wild-type cells following stimulation. Additional regulatory mechanisms may involve PRAS40, the negative regulator of mTORC1^43,44^, which can be phosphorylated by Akt to relieve its inhibition, further enhancing nuclear mTORC1 activity. Indeed, we showed that PRAS40 knockdown in cells expressing nuclear-localized Raptor significantly increased basal mTORC1 activity and diminished the PDGF-stimulated activity (Fig. 8a, b, d, e), suggesting that Akt-mediated suppression of PRAS40-inhibition of mTORC1 provides an additional mechanism for growth factor dependent regulation of nuclear mTORC1 activity. On the other hand, the direct and essential activator for mTORC1, Rheb GTPase, has been suggested to localize in the nucleus^7^, where relatively low levels of nuclear TSC2, the only well-established regulator and GAP of Rheb, is present. Thus, nuclear translocation of Raptor and phosphorylation of PRAS40, both regulated by Akt, are two major mechanisms that potentially regulate nuclear mTORC1 (Supplementary Fig. 12).

These findings provide the basis for future work to identify nuclear-specific mTORC1 substrates and elucidate the functions of nuclear mTORC1. Nuclear mTORC1 signaling has been suggested to promote transcription of metabolic genes^11,15,52^. For example, mTORC1 was shown to interact with the transcription factor yin-yang 1 (YY1) at promoter sites of the genes encoding PPARc coactivator-1 (PGC1a) and cytochrome c, and inhibition of mTORC1 by rapamycin resulted in a failure of YY1 to interact with and be coactivated by PGC-1a^52^. While we show in this study that nuclear Akt-STOPS suppresses pol III transcription, suggesting nuclear mTORC1 regulates pol III transcriptional activity, additional roles of nuclear mTORC1 and its nuclear-specific substrates have yet to be uncovered and identified. Diversity in the regulation of different pools of mTORC1 could lead to specificity in functional controls and also provide opportunities for selective targeting.

## Methods

### Constructs

To construct Akt-STOPS, AktAR2 in pcDNA3 (Addgene #64932) was cut with *BamH*I/*EcoR*I to replace the backbone with pRSetB to generate AktAR2 in pRSetB. AktAR2 in pRSetB was then cut with *BamH*I/*Sph*I to replace cerulean3 with mCherry. Then, this backbone was cut with *Sph*I/*Sal*I and ligated with a pair of annealed primers encoding Akt substrate recognition sequence derived from FoxO1 (PRPRSCTWPDPRPEF) with *Sph*I/*Sal*I restriction sites, followed by cutting with *Sac*I/*EcoR*I and ligation with a pair of annealed primers encoding PRPRSCTWPDPRPEF with *Sac*I/*EcoR*I restriction sites to generate the final Akt-STOPS in pRSetB. The construct was verified by sequencing after subcloning into a modified version of the mammalian expression vector pcDNA3′. Scramble peptide sequence was generated by randomizing Akt substrate recognition sequence derived from FoxO1 using peptidenexus.com. To generate mCherry tagged scramble peptide, mChe-SP, a series of primers encoding TPCPSEWPRRPRDPF were used following the above cloning scheme. The nuclear-targeted constructs, TORCAR-NLS, AktAR2-NLS, Akt-STOPS-NLS and SP-NLS, were constructed by tagging a nuclear localization signal (NLS) PKKKRKVEDA^53^ to the C terminus of TORCAR^23^, AktAR2^23^, Akt-STOPS and mChe-SP, respectively. The cytosolic targeted constructs, TORCAR-NES and AktAR2-NES, were generated by fusing a nuclear export signal (NES)^54^ to the C terminus of TORCAR and AktAR2. The plasma membrane-targeted constructs, PM-AktAR2 and PM-Akt-STOPS, were constructed by the addition of the N-terminal portion of the Lyn kinase (GCIKSKRKDKD) to the 5′ end of AktAR2 and Akt-STOPS, respectively^55^. H2A-mChe was constructed by subcloning with a *Hind*III/*BamH*I-digested PCR fragment encoding Histone 2A and a *BamH*I/*EcoR*I-digested PCR fragment with stop codon encoding mCherry. H2A-mChe-S6K1 and H2A-mChe-Raptor were generated by subcloning with a *BamH*I/*Sph*I-digested PCR fragment encoding mCherry and *Sph*I/*EcoR*I-digested PCR fragment with stop codon encoding S6K1 or Raptor, respectively. FKBP and FRB constructs were kindly provided by Dr. Takanari Inoue (Johns Hopkins University). PM-FRB was generated by the addition of the N-terminal portion of the Lyn kinase (GCIKSKRKDKD) to the 5′ end of FRB. FKBP with a flexible linker was added to the N-terminus of Akt-STOPS^56^ using *Hind*III and *BamH*I restriction sites. Primers are listed in Supplementary Table 1. All plasmids will be made available by direct request from the investigators, as well as through Addgene.

### Reagents

PDGF (P3201), insulin (I-5523), IBMX (I5879) and Gö6983 (G1918) were purchased from Sigma. Nuclear-ID Red (Enzo-52406) was purchased from Enzo Life Sciences. Torin1 was purchased from TOCRIS (#4247). Leucine O-methyl ester was purchased from NovaBiochem. Leptomycin B (LMB, AAJ63784EXG) and Importazole (IPZ, S8446) were purchased from Fisher Scientific. Forskolin (F9929) and rapamycin (R-5000) were purchased from LC Labs. Rapalog (AP21967, #635067) was purchased from Takara Bio. GDC-0068 (RG7440) was purchased from APExBIO. STOPS peptide (PRPRSCTWPDPRPE) was synthesized by Fmoc solid-phase peptide synthesis and purified by reverse phase HPLC (95.8% purity) with standard TFA removal by GenScript. Identity and purity were confirmed by HPLC-MS. pLKO mouse shRNA 1 raptor was purchased from Addgene (#21339).

### Cell culture, transfection, and starvation

NIH3T3 cells (CRL-1658, ATCC) were cultured in Dulbecco’s modified Eagle’s medium (11885, Gibco) supplemented with 10% calf serum (30-2030, ATCC) and 1% of penicillin-streptomycin (Sigma-Aldrich) and were routinely tested for mycoplasma contamination and found negative. For live-cell imaging, cells were plated onto sterile glass-bottomed 35-mm dishes (D35-14-1.5-N, CellVis) and grown to 40% confluency at 37 °C with 5% CO_2_. For immunofluorescence and proximity ligation assay, NIH3T3 cells were seeded into 18 mm coverslip at ~30% confluency in 35 mm dishes containing glass coverslips (12-541A, FisherBrand). For transfection, cells were either transfected with Lipofectamine 2000 (Invitrogen) or nucleofected using 4D-Nucleofector System (Core and X-unit, Lonza) with recommended kits. For serum starvation, cells were starved in serum-free DMEM for 24 hr. For serum and amino acid starvation (double starvation, referred as “DS”, and cells were double-starved if not otherwise indicated), cells were serum-starved for 24 h (DMEM without serum) followed by 2 h amino acid starvation in modified Hank’s balanced salt solution (1 × HBSS with 2 g/l glucose, pH 7.4, made from 10 × HBSS (14065, GIBCO)) at 37 °C.

### Differentiation and Nucleofection of 3T3-L1 Adipocytes

3T3-L1 preadipocytes were grown to confluency in 10% calf serum/DMEM and stimulated with induction media (DMEM containing 10% fetal bovine serum, 1 µg/ml insulin, 1 µM dexamethasone, and 0.5 mM 3-isobutyl-1-methylxanthine) at 2 days post-confluency. The medium was changed to insulin medium (1 µg/ml) 2 days after induction. Two days later, the medium was replaced with 10% fetal bovine serum/DMEM and then changed every 2 days. Nucleofection with H2A-TORCAR was carried out 10 days after full differentiation according to the manufacturer’s instructions (Lonza).

### Lentivirus production and cell transduction

Lentivirus was packaged in HEK293T cells. Specifically, HEK293T cells were co-transfected with lentiviral vector (containing sequences expressing shRNA targeting mouse raptor proteins)^57^ + psPAX2 + pMD2.G using PolyJet transfection reagent (SignaGen Laboratories, MD, USA) according to manufacturer’s instructions. After 48 hr the supernatants were collected, and then concentrated using Lenti-X concentrator (Takara Bio USA, Inc.) according to the manufacturer’s instructions. The concentrated virus was stored at −80 °C. For transduction, NIH3T3 cells were seeded, and concentrated solutions containing lentiviral particles were added into the cell culture medium. After 48 hr, cells were passed in fresh growth medium and were treated with puromycin (2 µg/mL) to select transduced cells. Cells were maintained in selection medium for one week and samples were collected for western blot and immunofluorescence staining^57^.

### Immunoblotting

Cells were washed with ice-cold PBS and then lysed in RIPA lysis buffer containing protease inhibitor cocktail, 1 mM PMSF, 1 mM Na_3_VO_4_, 1 mM NaF, and 25 nM calyculin A. Total cell lysates were incubated on ice for 30 min and then centrifuged at 4 °C for 20 min. Total protein was separated via 4–15% SDS-PAGE and transferred to PVDF membranes. The membranes were blocked with TBS containing 0.1% Tween-20 and 5% bovine serum albumin and then incubated with primary antibodies overnight at 4 °C. After incubation with the appropriate horseradish peroxidase-conjugated secondary antibodies, the membranes were developed using horseradish peroxidase-based chemiluminescent substrate (34579 and 34076, Thermoscientific). The intensity of the bands was quantified with ImageJ 1.52s software. The following primary antibodies were used for immunoblotting: p-4EBP1 (T37/46) (#2855), 4EBP1 (#9452), p-S6K1 (T389) (#9205), S6K1 (#9202), p-GSK3β (S9) (#9322), p-PRAS40 (T246) (#2997), PRAS40 (#2691), p-NDRG1 (S330) (#3506), NDRG1 (#5196), p-rpS6 (S240/244) (#5364), rpS6 (#2317), p-FoxO1 (S256) (#9461), FoxO1 (#2880), p-RanBP3 (S58) (#9380) and tubulin (#2146) antibodies from Cell Signaling Technology, GSK3β (#610201) antibody from BD Bioscience, RanBP3 (700076) antibody from Invitrogen, Raptor antibody (20984-1-AP) from Proteintech, and RFP antibody (A00682-100) from GenScript. The horseradish peroxidase-labeled goat anti-rabbit (PI31460) or anti-mouse (PI31430) secondary antibodies were purchased from Pierce.

### Protein purification

The catalytic domain of Akt3 (residues N118 to E479) was subcloned into p3XFlag-CMV-10 (Sigma) and transfected into 3 × 15 cm dishes of HEK293T cells using Effectene (QIAGEN). Flag-Akt3 catalytic domain (Akt3-Cat) was purified as described^58^. Briefly, cells expressing Flag-tagged Akt3-Cat were lysed in 20 mM Tris (pH 7.5), 150 mM NaCl, 1 mM EDTA, 1 mM EGTA, 1% Triton X-100, 2.5 mM sodium pyrophosphate, 1 mM β-glycerophosphate, 1 mM Na_3_VO_4_, 1 mM DTT, 1 mM PMSF, 10 μg/ml leupeptin, 2 μg/ml pepstatin, 10 µg/ml aprotinin. The soluble cell lysate was incubated with anti-FLAG M2 beads (Sigma) for 1 h at 4 °C. After 4 washes total, Akt3-Cat protein was eluted in 50 mM HEPES, pH 7.4, 100 mM NaCl, 1 mM DTT, 5 mM β-glycerophosphate, 0.1 mM Na_3_VO_4_, 0.01% NP40 (Igepal CA630), 10% glycerol containing 0.5 mg/mL 3xFLAG Peptide (Sigma)^58^. Protein was quantified using Coomassie (Bradford) Protein Assay (Thermofisher) and stored at −80 °C until use. Human PKCβII was cloned into the pFastBac HT/B vector (ThermoFisher) that was modified with a GST tag. PKCβII protein was purified as described^42^. Briefly, Sf-21 insect cells expressing GST-PKCβII protein were lysed in 50 mM HEPES, pH 7.5, 1 mM EDTA, 100 mM NaCl, 0.1% Triton X-100, 100 µM PMSF, 1 mM DTT, 2 mM benzamidine, 50 µg/mL leupeptin, and 1 µM microcystin. The soluble cell lysate was incubated with glutathione Sepharose beads for 30 min at 4 °C. After washes, GST-PKCβII was eluted in 50 mM HEPES, pH 7.5, 1 mM EDTA, 100 mM NaCl, 0.1 mg/mL BSA, and 1 mM DTT with 10 mM glutathione. Pure protein was quantified by Coomassie gel using BSA as controls and then stored in 50% glycerol at −20 °C until use.

### In vitro kinase assays

For Akt3 catalytic domain assays, 50 ng of pure protein was used per time point. 80 μl reactions were carried out in 20 mM HEPES, pH7.5, 2 mM MgCl_2_, 2 mM DTT, 100 μM ATP, 0.1 mg/ml BSA, ~ 50 μCi/ml γ-^32^P-ATP (Perkin Elmer) with 0, 3, 10, 30, 100 and 300 μM Akt-STOPS peptide (GenScript) for 10 min at 30 °C. Reactions were quenched by addition 25 μl 0.1 M ATP, 0.1 M EDTA, pH 8 and spotted on P81 paper (Whatman), washed 4 times with 500 ml 0.4% phosphoric acid and counted in a scintillation counter (Beckman). 30 μM Crosstide peptide was used to confirm Akt3 catalytic domain activity; from this, the specific activity of the preparation was determined to be 380 nmol/min/mg. For PKCβII kinase assays, 50 ng of pure protein was used per time point. 80 μl reactions were carried out in 20 mM HEPES, pH 7.4, 2 mM DTT, 5 mM MgCl_2_, 100 µM ATP, ~1 µCi gamma-32P-ATP, 140 µM/3.8 µM phosphatidylserine/diacylglycerol membranes, 100 µM Ca^2+^. Stocks of Akt-STOPS peptide were prepared for final concentrations of 0, 3, 10, 30, 100 and 300 μM, and the kinase reactions were carried out for 10 min at 30 °C^59^. Reactions were quenched by addition 25 μl 0.1 M ATP, 0.1 M EDTA, pH 8 and spotted on P81 paper (Whatman), washed 4 times with 500 ml 0.4% phosphoric acid and counted in a scintillation counter (Beckman). 100 μM of MARCKS peptide was used to determine the activity of the PKCβII preparation; from this, the specific activity was determined to be 580 nmol/min/mg.

### Nuclear Fractionation

NIH3T3 cells were seeded in a 10 cm dish and grown to 80% confluency. Cells were either left untreated or treated for 30–40 min with 50 ng/ml PDGF. Cells were washed twice with ice-cold 1× PBS and harvested in 1× PBS. Following centrifugation for 5 min at 2348 × *g* at 4 °C, cell pellet was resuspended in 150 µL of 1× hypotonic lysis buffer (20 mM HEPES, pH 7.4, 10 mM NaCl, 3 mM MgCl_2_, Roche protease cocktail inhibitors, 1 mM PMSF, 1 mM Na_3_VO_4_, 1 mM NaF, and 25 nM calyculin A), and incubated for 1 h to allow for cell lysis. Cells were passed through a 27 1/2 G needle 15 times to maximize lysis. BCA quantification was performed, and protein levels were normalized to the lowest protein concentration (1–2 mg/ml). To extract nuclear content, the membrane proteins were solubilized by adding NP-40 at a final concentration of 0.5% and incubated for 1 h on ice. All samples were centrifuged at 2348 × *g* for 10 min, nuclear pellets were saved and supernatant was centrifuged a second time to remove any remaining nuclear impurities. Nuclear pellets were washed twice, combined accordingly in 150 µL of hypotonic buffer, sonicated to solubilize pellet. Samples were prepared with Laemmli sample buffer with 0.1 mM DTT. Equal protein amounts within respective fractions were loaded^60^ and assessed via SDS-PAGE on 9% gels or gradient gels. Proteins were transferred to PVDF membranes. Membranes were blocked for 1 h in 4% BSA in 1× TBST and probed overnight in 4% BSA in 1× TBST for mouse anti-p84 (GTX70220, GeneTex), mouse anti-GAPDH (GTX627408, GeneTex), p-mTOR (S2448) (#2971, Cell Signaling), mTOR (#2972, Cell Signaling), Raptor (20984-1-AP, Proteintech), p-PRAS40 (T246) (#2997, Cell Signaling), and PRAS40 (#2691, Cell Signaling). Blots were incubated in 5% milk in 1× TBST for 1 h at room temperature and appropriate HRP-conjugated secondary antibodies. Blots were subjected to chemiluminescence, blots were analyzed by densitometry in Image J. For quantification, levels of target proteins in either cytoplasmic or nuclear fractions were normalized to each respective control (GAPDH for cytoplasmic fractions and p84 for nuclear fractions), and the changes between non-treated and treated samples were compared by setting the non-treated sample as 1. In this protocol, we loaded equal protein amounts for each respective fractions, and normalized the levels of the target proteins within respective fractions to a fraction marker (GAPDH and p84 for cytoplasmic and nuclear fractions, respectively), to cancel out the potential loading error^61–63^. This protocol is suited for comparing protein levels in the nuclear fraction before and after PDGF treatment but not designed to compare protein abundance between the cytoplasmic and nuclear fractions^60^.

### Proximity ligation assay

PLA experiments were performed using the Duolink in situ red starter kit for proximity ligation assays (Sigma Aldrich, DUO92101) following the manufacturer’s protocol. Briefly, cells grown on coverslips were fixed and permeabilized as in the immunofluorescence experiments then blocked with blocking buffer supplied with the kit at 37 °C for 1 h. Cells were incubated with the primary antibody (mouse anti-mTOR, 66888-1-Ig, Proteintech, 1:100, and rabbit anti-Raptor, 20984-1-AP, Proteintech, 1:100) overnight at 4 °C, and then with the provided secondary antibody (conjugated with nucleotides) for 1 h at 37 °C with washes after each step. Ligation of the nucleotides and amplification of the strand occurred sequentially by incubating cells with first ligase and then polymerase and detection solution. PLA experiments with mTOR antibodies from different species (mouse anti-mTOR, 66888-1-Ig from Proteintech, 1:100, rabbit anti-mTOR, #2983 from Cell Signaling, 1:100) were used as positive controls, and experiments with mouse anti-mTOR only or mouse anti-mTOR with normal rabbit IgG (#2729, Cell Signaling) provided negative controls.

### Immunofluorescence

Cells were washed 3 times with PBS and fixed with 4% paraformaldehyde in PBS (15710 S, Electron Microscopy Sciences) for 30 min at room temperature. Cells were then washed 3 times with PBS and permeabilized with PBS containing 0.1% Triton X-100 for 15 min at room temperature. Following 1-h incubation in blocking buffer (PBS containing 0.1% Triton X-100 and 5% BSA) at room temperature, coverslips were incubated for 12 h at 4 °C in primary antibody diluted in blocking buffer. The following primary antibodies were used for immunofluorescence: Raptor (20984-1-AP, Proteintech, 1:100), mTOR (#2983, Cell Signaling, 1:100), p-PRAS40 (T246) (#2997, Cell Signaling, 1:100), PRAS40 (ThermoFisher, AHO1031, 1:100), RanBP3 (Invitrogen, 700076, 1:100). Following three 5 min washes in PBS, coverslips were incubated for 1 h at room temperature in the dark in secondary antibody (anti-rabbit Alexa Fluor 488, A11006 (1:1000), anti-mouse Alexa Fluor 568, A11004 (1:1000), Life Technologies/Molecular Probes) diluted in blocking buffer containing 0.1% Triton X-100. Following three 5 min washes with PBS, coverslips were mounted in Prolong Glass antifade Mountant with NucBlue (P36981, Invitrogen).

### Microscopy and image analysis

For live-cell imaging, cells were washed once with modified Hank’s balanced salt solution (1 × HBSS with 2 g/l glucose, pH 7.4, made from 10 × HBSS (14065, GIBCO)) and imaged in the dark at room temperature. Images were acquired on a Zeiss Axio Observer Z1 microscope equipped with a 40x/1.3NA objective and Photometrics Evolve 512 EMCCD. Dual-emission ratio imaging was performed with a 420DF20 excitation filter, a 450DRLP dichroic mirror, and two emission filters, 475DF40 and 535DF25 for CFP and YFP, respectively. For RFP, a 568DF55 excitation filter, a 600DRLP dichroic mirror, and a 653DF95 emission filter were used. Exposure times were 50–500 ms, and images were taken every 30 s. Imaging data was analyzed with Metafluor 7.7 software (Molecular Device). Fluorescence images were background-corrected by deducting the background (regions with no cells) from the emission intensities of CFP or YFP. Regions of interest (ROIs) at the plasma membrane were used for analysis for PM-AktAR2. The ratios of emissions (cyan-to-yellow for TORCAR related imaging and yellow-to-cyan for AktAR2 related imaging) were then calculated at different time points. The ratio was normalized to the average ratio of timepoints before the addition of drugs.

For PLA, the two-color (blue and red, excitation 405/561 nm, emission filter BP420-480 + LP605) confocal images were acquired by a Zeiss LSM880 microscope equipped with a Plan-Apochromat 63X/1.4 oil immersion objective and an airyscan super-resolution detector, using Zen Black Zeiss software for acquisition and processing. The z-stack containing nine optical slices with a thickness of 0.385 μm for each slice was acquired for both channels. The images were 1000 × 1000 pixels with a pixel size of 137 nm. Identical settings were kept constant within a batch of experiments. For each condition, four different fields and 20 to 50 cells were analyzed. For quantitative analyses, the central slice of a z-stack containing 9 slices (0.385 μm each) was used, and the number of dots were counted within the nucleus using CellProfiler 3.0.0 software (Broad Institute)^64^.

For quantifying Raptor translocation, the two-color (blue and green, excitation 405/488 nm, emission filter BP420-480 + BP495-550) confocal images were acquired by a Zeiss LSM880 microscope as described above, except the images were 3812 × 3812 pixels with a pixel size of 35 nm. Identical settings were kept constant within a batch of experiments. For each condition, three to seven different fields and 20 to 30 cells were analyzed per condition. For measuring Akt-STOPS effects on Raptor translocation, the three-color (green, red and blue) images were acquired by Zeiss Axio Observer Z1 microscope with a 40x/1.3NA objective and Photometrics Evolve 512 EMCCD. For DAPI and mCherry imaging, settings were the same as described above in the live-cell time lapse imaging section. For AF488, HQ480/30x excitation filter and HQ535/45 m emission filter were used. For each condition, 10–20 different fields and 100–600 cells were analyzed per condition.

The nuclear to cytosol ratio was quantified using CellProfiler (Broad Institute) software^64^. Briefly, images were thresholded in each color channel to determine the nuclear and cytoplasmic areas outside of the nucleus. The mean intensity of nuclear fluorescence was then measured as the average pixel intensity within the nuclear area, and the mean intensity of cytoplasmic fluorescence was determined by the average pixel intensity in the non-nuclear cytoplasmic area. Lastly, the nuclear to cytoplasmic ratio was calculated as the mean intensity of nuclear fluorescence divided by the mean intensity of cytoplasmic fluorescence. For cells transfected with H2A-mChe or H2A-Akt-STOPS, the nuc/cyto ratio of Raptor immunostaining was calculated only for cells showing red fluorescence above threshold. For validation of Raptor antibody, the mean intensity of entire cell including cytosol and nucleus was measured.

### Time course curve fitting

Using CFTool (3.4.1) in MatLab 8.3.0.532 (R2014A), the reporter response curves were fitted with an empirical sigmoidal funcation (Eq. 1), where F_0_ is the response at the baseline, F_max_ is the response at the plateau of the progress curve, *t*_*0*_ is the sigmoid’s midpoint, and *k* is an apparent rate^65^. The lag time *t*_*lag*_ is estimated by extrapolating the tangent at the inflection time point to the initial baseline, and is then calculated by Eq. (2).

1 $$F(t) = \frac{{F_{\max }}}{{1 + v{\mathop{\rm{e}}\nolimits} ^{ - k(t - t_0)}}} + F_0,$$

2 $$t_{{\rm{lag}}} = t_0 + \frac{{\ln (v) - 2}}{k}.$$

### RT-qPCR analysis

RNA was extracted from cultured cells by using TRIzol (#15596018, Invitrogen, Carlsbad, CA). Total RNA was reverse-transcribed by using Takara (#RR036A-1, Clontech Laboratories, Mountain View, CA), then underwent quantitative real-time PCR (qPCR) with SYBR Green (#1725120, Bio-Rad, Hercules, CA). The relative level of mRNA was calculated by the ΔΔCq method with β-actin as an internal control.

### Statistics and reproducibility

All experiments were independently repeated as noted in the figure legends. All replication attempts were successful. The data were analyzed using GraphPad Prism 6. For Gaussian data, pairwise comparisons were performed using two-sided Student’s *t* test or Welch’s unequal variance *t*-test, and comparisons among three or more groups were performed using ordinary one-way ANOVA followed by Dunnett’s test or Tukey’s test for multiple comparisons. For comparisons in Supplementary Fig. 10b, statistical analyses were performed using two-way ANOVA followed by Dunnett’s multiple comparisons tests. Statistical significance was set at *P* < 0.05. **** indicates a *p*-value < 0.0001; *** indicates a *p*-value between 0.0001 to 0.001; ** indicates a *p*-value between 0.001 to 0.01; * indicates a *p*-value between 0.01 to 0.05; ns, *p* > 0.05, not significant. n numbers, as indicated in figure legends and the main text, represent number of cells. All data are presented as mean ± s.e.m. and violin plots depict the median and quartiles, as indicated in the figure legends.

### Reporting summary

Further information on research design is available in the Nature Research Reporting Summary linked to this article.

## Supplementary information

Supplementary Information

Reporting Summary

## Data Availability

The authors declare that all the data supporting the findings of this study are available within the paper and its supplementary information files or from the corresponding author upon reasonable request. Source data are provided with this paper.

## Source data

Source Data
