# Supplementary material for: Location-specific inhibition of Akt reveals regulation of mTORC1 activity in the nucleus
Source: Nat Commun. 2020 Nov 30;11:6088. doi: 10.1038/s41467-020-19937-w (PMC7705703; doi:10.1038/s41467-020-19937-w)
Supplement: Supplementary file 1 — Supplementary Information [file 41467_2020_19937_MOESM1_ESM.pdf]

Supplementary Information

Location-specific inhibition of Akt reveals regulation of mTORC1 activity in the nucleus

Xin Zhou[1], Yanghao Zhong[1,2], Olivia Molinar-Inglis[1], Maya T. Kunkel[1], Mingyuan Chen[3], Tengqian Sun[1], Jiao Zhang[4], John Y.-J. Shyy[4], JoAnn Trejo[1], Alexandra C. Newton[1], and Jin Zhang[1,3,5]*

[1] Department of Pharmacology, University of California, San Diego, La Jolla, CA, USA
[2] Biomedical Sciences Graduate Program, University of California, San Diego, La Jolla, CA, USA
[3] Department of Bioengineering, University of California, San Diego, La Jolla, CA, USA
[4] Division of Cardiology, Department of Medicine, University of California San Diego, La Jolla, CA USA
[5] Department of Chemistry & Biochemistry, University of California, San Diego, La Jolla, CA, USA

* e-mail: jzhang32@ucsd.edu

Supplementary Figure 1

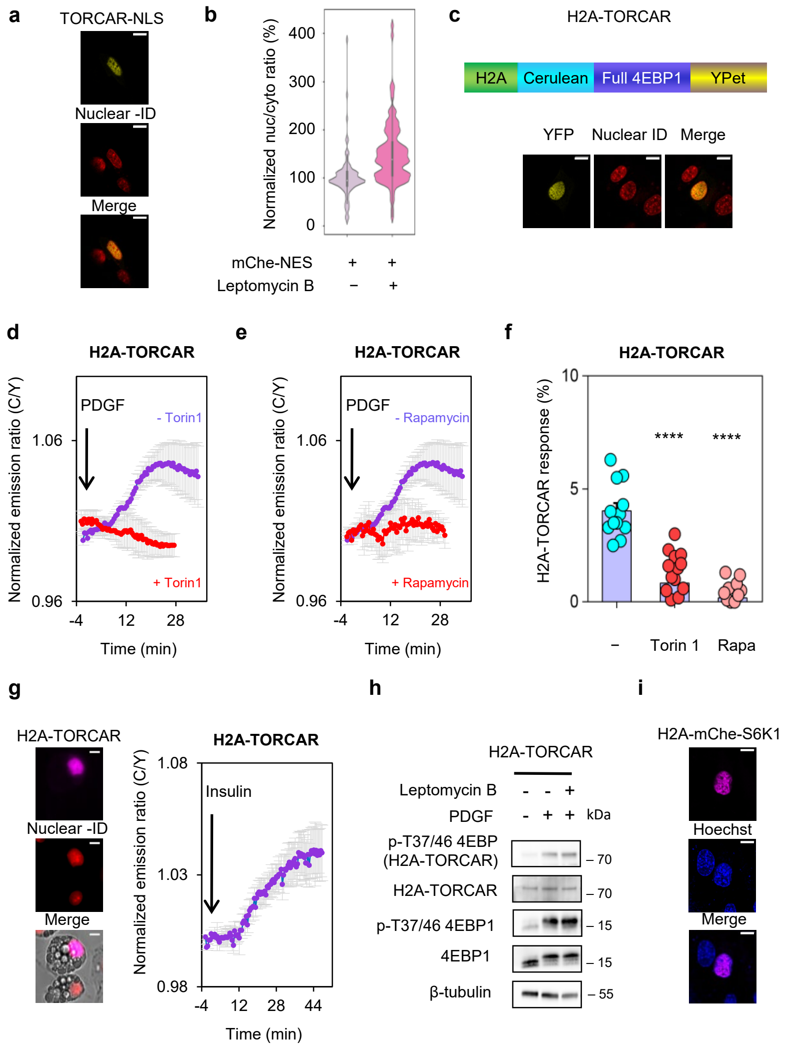

Supplementary Fig. 1 Nuclear mTORC1 activity is present. a. Double-starved NIH3T3 cells expressing TORCAR-NLS (YFP) stained with nuclear-ID. Scale bar = 10 µm. Representative of three experiments. **b**. Quantification of Nuc/Cyto ratio of mCherry fluorescence in double-starved NIH3T3 cells expressing mChe-NES without (n = 122 cells) or with treatment of leptomycin B (100 nM, 30 min, n =112 cells). Pooled from three experiments. Boxes indicating interquartile range and mid-points indicating median. Unpaired two-tailed student's t-test. ****, p=3 x 10^{-11}. **c**. Domain structure of nuclear targeted TORCAR (H2A-TORCAR), and its expression in NIH3T3 cells. Scale bar = 10 µm. Representative of three experiments. **d**. Average time courses of normalized emission ratio (Cyan/Yellow) in double-starved NIH3T3 cells expressing H2A-TORCAR stimulated with 50 ng/ml of PDGF without (blue trace, n = 13 cells from 4 experiments) or with pretreatment of Torin1 (1µM , 10 min) (red trace, n = 16 cells from 3 experiments). **e**. Average time courses of normalized emission ratio (Cyan/Yellow) in double-starved NIH3T3 cells expressing H2A-TORCAR stimulated with 50 ng/ml of PDGF without (blue trace, n = 13 cells from 4 experiments) or with pretreatment of rapamycin (50 µM, 10 min) (red trace, n = 12 cells from 3 experiments). **f**. Summary of responses of H2A-TORCAR in PDGF-treated double-starved NIH3T3 cells without pretreatment, with pretreatment of Torin1 (****, p=1 x 10^{-18}) or rapamycin (****, p=2 x 10^{-17}), as in panel d and e. Error bar represents mean ± s.e.m. One-way ANOVA followed by Dunnett's tests. Source data are provided as a Source Data file. **g**. Average time courses of normalized emission ratio (Cyan/Yellow) in double-starved 3T3L1 adipocytes expressing H2A-TORCAR stimulated with 400 ng/ml of insulin. n = 3 experiments. Left: 3T3L1 adipocytes expressing H2A-TORCAR were stained with nuclear-ID. Representative of three experiments. Scale bar = 10 µm. **h**. Western blot analysis of double-starved NIH3T3 cells expressing H2A-TORCAR treated with leptomycin B (100 nM, 30 min) prior to PDGF stimulation(50 ng/ml, 30 min). Representative of three independent experiments. Full blots are shown in Supplementary Fig. 14. **i**. Double-starved NIH3T3 cells expressing H2A-mChe-S6K1 show the nuclear localization. Representative of three experiments. Scale bar = 10 µm.

Supplementary Figure 2

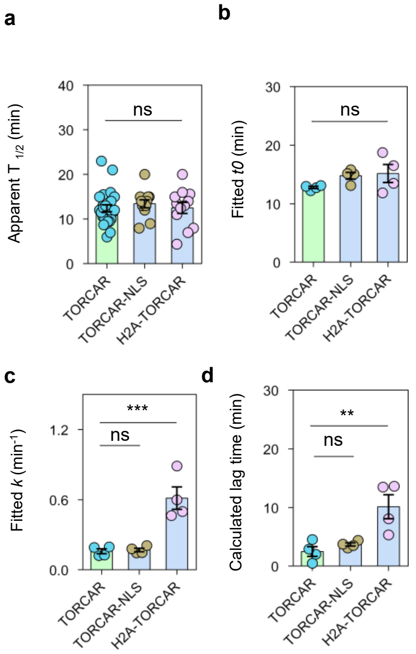

Supplementary Fig. 2 Comparison of reporter kinetics. a. Comparison of apparent half-time ($T_{1/2}$) of diffusive TORCAR, and two nuclear TORCARs. n = 25, 12, 12 cells for TORCAR, TORCAR-NLS, and H2A-TORCAR, respectively. Error bar represents mean ± s.e.m. Statistical analyses were performed using ordinary one-way ANOVA followed by Dunnett's multiple comparisons tests. TORCAR vs. TORCAR-NLS, ns, not significant, p=0.3; TORCAR vs. H2A-TORCAR, ns, not significant, p=0.2. Source data are provided as a Source Data file. **b**. Comparison of fitted parameter, t_0 (see Eq. (1) in Methods). n = 4, 4 and 4 for TORCAR, TORCAR-NLS, H2A-TORCAR, respectively. Error bar represents mean ± s.e.m. Ordinary one-way ANOVA followed by Dunnett's multiple comparisons tests. TORCAR vs. TORCAR-NLS, ns, not significant, p=0.2; TORCAR vs. H2A-TORCAR, ns, not significant, p=0.1. **c**. Comparison of fitted parameter, *k* (see Eq. (1) in Methods). n = 4, 4 and 4 for TORCAR, TORCAR-NLS, H2A-TORCAR, respectively. Error bar represents mean ± s.e.m. Ordinary one-way ANOVA followed by Dunnett's multiple comparisons tests. TORCAR vs. TORCAR-NLS, ns, not significant, p=0.9; TORCAR vs. H2A-TORCAR, ***, $p=3 \times 10^{-4}$. Source data are provided as a Source Data file. **d**. Comparison of fitted parameter, t_{lag} (see Eq. (2) in Methods). n = 4, 4 and 4 for TORCAR, TORCAR-NLS, H2A-TORCAR, respectively. Error bar represents mean ± s.e.m. Ordinary one-way ANOVA followed by Dunnett's multiple comparisons tests. TORCAR vs. TORCAR-NLS, ns, not significant, p=0.5; TORCAR vs. H2A-TORCAR, ***, $p=2 \times 10^{-3}$. Source data are provided as a Source Data file.

Supplementary Figure 3

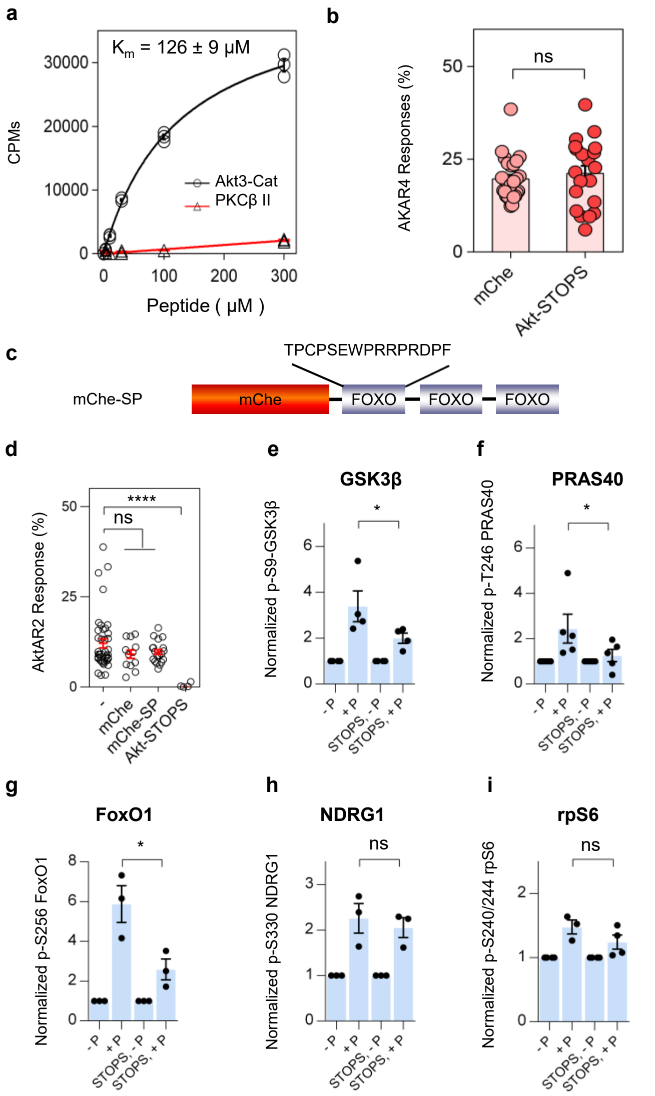

Supplementary Fig. 3 Characterization of Akt-STOPS. a. *In vitro* characterization of K_m of Akt-STOPS using Akt3 catalytic domain or PKCβII. K_m of 126 ± 9 µM was derived for Akt3 catalytic domain. **b**. Responses of Hek293T cells expressing AKAR4 with mCherry (19.7 ± 1.1%, n = 26 cells from 4 experiments) or Akt-STOPS (21.2 ± 2.0%, n = 20 cells from 4 experiments) to 50 µM of Forskolin and 100 µM of IBMX. Error bar represents mean ± s.e.m. P value was determined by unpaired two-tailed student's t-test with Welch's correction. Source data are provided as a Source Data file. **c.** Domain structure of mCherry tagged scramble peptide (mChe-SP). **d**. Summary of responses of PDGF-treated serum-starved NIH3T3 cells expressing AktAR2 without or with co-expression of mCherry, mChe-SP or Akt-STOPS. The responses of AktAR2 alone (-), with expression of mCherry (mChe), with expression of mChe-SP (mChe-SP), and with expression of Akt-STOPS (Akt-STOPS) are 12 ± 1% (n = 41 cells from 4 experiments), 9 ± 1% (n = 12 cells from 3 experiments), 10 ± 1% (n = 19 from 5 experiments), and 0 ± 3% (n = 7 from 7 experiments), respectively. All data are mean ± s.e.m. One-way ANOVA followed by Dunnett's multiple comparisons tests. - vs. mChe, ns, not significant, p=0.1; - vs. mChe-SP, ns, not significant, p=0.2; - vs. Akt-STOPS, ****, $p=9 \times 10^{-6}$. Source data are provided as a Source Data file. **e-i**. Quantification of relative phosphorylation of GSK3β (**e**, *, p=0.046), PRAS40 (**f**, *, p=0.027), FoxO1 (**g**, *, p=0.016), NDRG1 (**h**, ns, not significant, p=0.63), and rpS6 (**i**, ns, not significant, p=0.046) in western blot experiments described in Fig.2c after normalizing to the corresponding unstimulated conditions. Values are means ± s.e.m. Data were representative of 4, 5, 3, 3, 3 experiments for **e-i**, respectively. P value was determined by paired two-tailed student's t-test.

Supplementary Figure 4

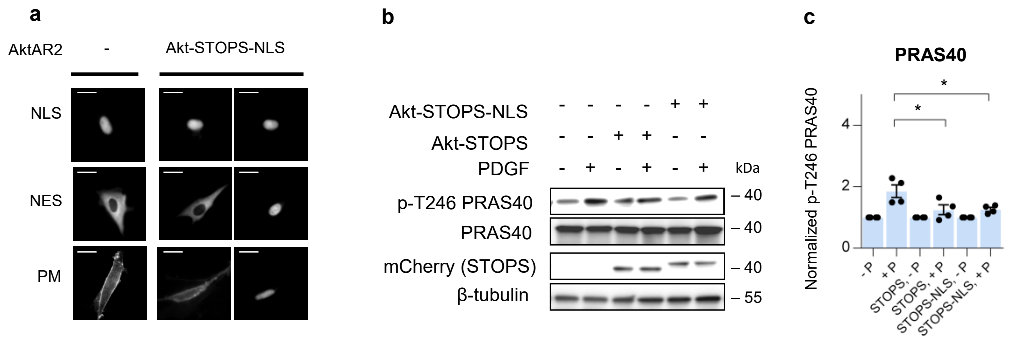

Supplementary Fig. 4 Characterization of Akt-STOPS-NLS. a. Representative images showing serum-starved NIH3T3 cells expressing AktAR2-NLS, NES, and PM alone (left) or with co-expression of Akt-STOPS-NLS (right). Scale bar = 10 μm. **b**. Western blot analysis of serum-starved NIH3T3 cells expressing Akt-STOPS or Akt-STOPS-NLS treated with PDGF (50 ng/ml) for 30 min. Representative of four repeats. Full blots are shown in Supplementary Fig. 14. **c**. Quantification of relative phosphorylation of PRAS40 in western blot experiments described in Supplementary Fig. 4b after normalizing to the corresponding unstimulated conditions. Data is representative of 4 independent experiments. Values are means ± s.e.m. P value was determined by paired two-tailed t-test. +P vs. STOPS, +P, *, p=0.027; +P vs. STOPS-NLS, +P, *, p=0.033.

Supplementary Figure 5

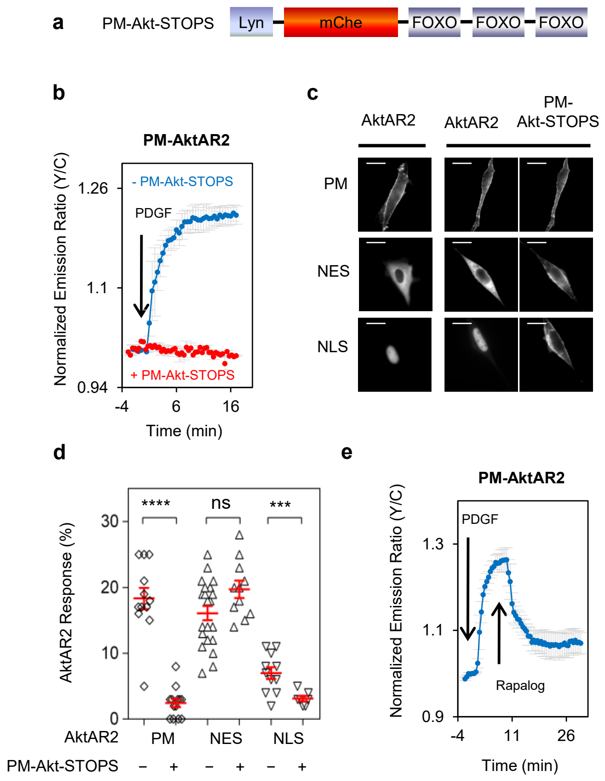

Supplementary Fig. 5 Characterization of plasma membrane Akt-STOPS. a. Domain structure of plasma membrane Akt-STOPS (PM-Akt-STOPS). **b**. Average time course of normalized emission ratio (Yellow/Cyan) in serum-starved NIH3T3 cells expressing PM-AktAR2 stimulated with 50 ng/ml of PDGF without (blue trace, n = 17 cells) or with expression of PM-Akt-STOPS (red trace, n =13 cells). Curves are representative of and pooled from 3 and 3 experiments, respectively. **c**. Representative images showing serum-starved NIH3T3 cells expressing PM-AktAR2, AktAR2-NLS and AktAR2-NES alone (left) or with co-expression of PM-Akt-STOPS (right). Scale bar = 10 µm. **d**. Summary of responses of PDGF-treated serum-starved NIH3T3 cells expressing subcellular targeted AktAR2 with or without co-expression of PM-Akt-STOPS. Data are presented as mean ± s.e.m. The responses of PM-AktAR2 without and with PM-Akt-STOPS are 18 ± 1% (n = 17 cells from 3 experiments) and 2.8 ± 0.7% (n = 13 cells from 3 experiments), respectively, unpaired two-tailed student's t-test with Welch's correction, ****, $p=7 \times 10^{-11}$; the responses of AktAR2-NES without and with PM-Akt-STOPS are 16.1 ± 1.1% (n =21 cells from 5 experiments) and 20 ± 1% (n =11 cells from 4 experiments), respectively, unpaired two-tailed student's t-test, ns, not significant, p=0.1; the responses of AktAR2-NLS without and with PM-Akt-STOPS are 7.0 ± 0. 8% (n = 11 cells from 4 experiments) and 3.7 ± 0.4% (n = 19 cells from 4 experiments), respectively, unpaired two-tailed student's t-test, ***, $p=3 \times 10^{-4}$. Source data are provided as a Source Data file. **e**. Average time course of normalized emission ratio (Yellow/Cyan) in serum-starved NIH3T3 cells expressing PM-AktAR2, PM-FRB, and mCherry-FKBP-STOPS showed a response to PDGF stimulation (50 ng/ml) that was reversed by addition of rapalog (3 µM). n = 5 cells from 3 experiments.

Supplementary Figure 6

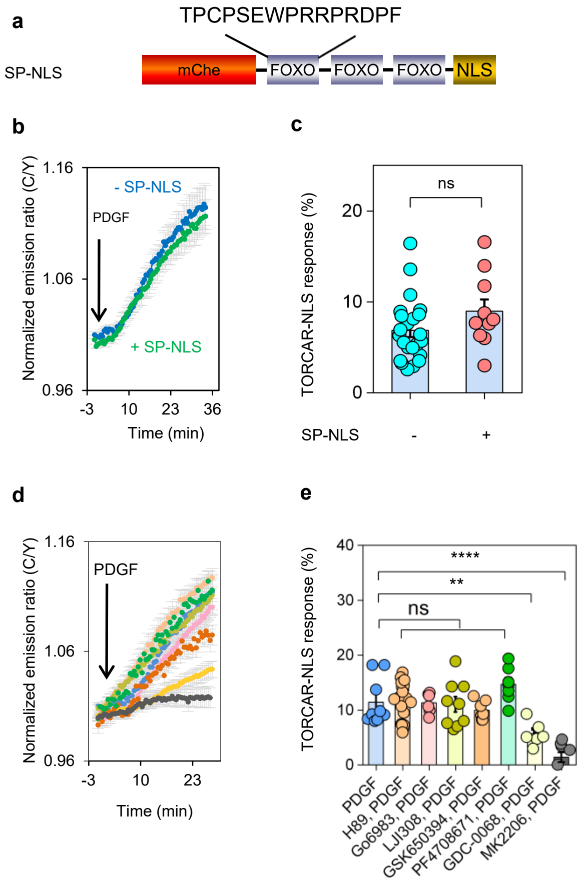

Supplementary Fig. 6 Nuclear mTORC1 activity is specifically suppressed by Akt inhibition. a. Domain structure of nuclear targeted scramble peptide (SP-NLS). **b**. Average time course of normalized emission ratio (Cyan/Yellow) in double-starved NIH3T3 cells expressing TORCAR-NLS stimulated with 50 ng/ml of PDGF without (blue trace, n = 23 cells from 4 experiments) or with expression of nuclear targeted scramble peptide (SP-NLS) (green trace, n = 10 cells from 5 experiments). **c**. Summary of responses of PDGF-treated double-starved NIH3T3 cells without or with expressing nuclear targeted scramble peptide (SP-NLS). Error bar represents mean ± s.e.m. P value was determined by unpaired two-tailed student’s t-test. ns, not significant, p=0.1. Source data are provided as a Source Data file. **d**. Average time course of normalized emission ratio (Cyan/Yellow) in double-starved NIH3T3 cells expressing TORCAR-NLS stimulated with 50 ng/ml PDGF without (blue trace) or with 5 min pretreatment of PKA inhibitor H89 (10 µM, light orange), PKC inhibitor Gö 6983 (1 µM, light pink), RSK inhibitor LJI308 (1 µm, lime green), S6K inhibitor PF4708671 (2 µM, green), SGK inhibitor GSK650394(2 µM, orange) , GDC-0068 (1 µM, dark yellow) or Akt inhibitor MK2206 (1 µM, grey). Curves are representative of and pooled from 4, 6, 4, 6, 5, 4, 5 and 4 experiments, respectively. **e**. Summary of responses of TORCAR-NLS in PDGF-treated double-starved NIH3T3 cells without (n = 9 cells from 4 experiments) or with pretreatment of H89 (n = 19 cells from 6 experiments, ns, not significant, p=0.9), Gö 6983 (n = 6 cells from 4 experiments, ns, not significant, p=0.9), LJI308 (n = 9 cells from 6 experiments, ns, not significant, p=0.8), GSK650394 (n = 7 cells from 5 experiments, ns, not significant, p=0.4), PF4708671 (n = 8 cells from 4 experiments, ns, not significant, p=0.1), GDC-0068 (n = 6 cells from 5 experiments, **, p=1 x 10^{-3}), or MK2206 (n = 8 cells from 4 experiments, ****, p=2 x 10^{-8}). Data are presented as mean ± s.e.m. Statistical analyses were performed using ordinary one-way ANOVA followed by Dunnett's multiple comparisons tests. Source data are provided as a Source Data file.

Supplementary Figure 7

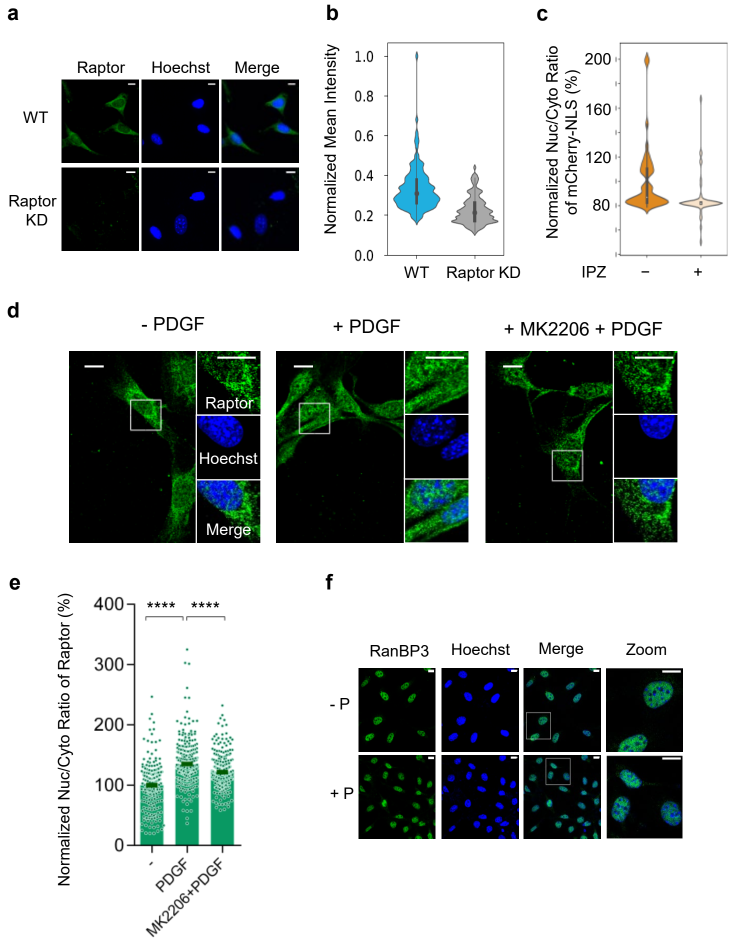

Supplementary Fig. 7 Akt facilitates nuclear translocation of Raptor. a. Validation of Raptor antibody. Representative images of Raptor immunostaining in WT or Raptor knockdown NIH3T3 cells. Representative of two independent experiments. Scale bar = 10 µm. **b**. Quantification of mean intensity of Raptor immunostaining per cell in wildtype and Raptor knockdown cells. p=1.7 x 10^{-20}. Boxes indicating interquartile range and mid-points indicating median. n = 137 and 147 cells for WT and Raptor KD from 3 experiments, respectively. **c**. Validation of importazole treatment. Quantification of Nuc/Cyto ratio of mCherry fluorescence in double-starved NIH3T3 cells expressing mChe-NLS without (n = 45 cells) or with importazole (100 µM) treatment for 1 hr (n =59 cells). Data are pooled from three experiments. Boxes indicating interquartile range and mid-points indicating median. P value was determined by unpaired two-tailed student's t-test. ***, p=6.79 x 10^{-4}. **d**. Representative confocal images of immunostaining of endogenous Raptor in double-starved NIH3T3 cells (- P), 30 min following PDGF stimulation (+ P), and pretreatment with 1 µM MK-2206 for 10 min followed by 30 min treatment with PDGF. The middle slices of confocal z-stacks were shown to observe the Raptor staining in the nucleus (Green). Representative of three independent experiments. Scale bar = 10 µm. **e.** Quantification of mean nuclear intensity/mean cytosol intensity ratio (Nuc/Cyto ratio) of Raptor immunostaining per cell. n = 341, 373, 371 cells. All data are mean ± s.e.m. Ordinary one-way ANOVA followed by Dunnett's multiple comparisons tests. - PDGF vs. + PDGF, ****, p=3 x 10^{-46}; + PDGF vs. + MK + PDGF, ****, p=4 x 10^{-9}. Source data are provided as a Source Data file. **f.** Localization of RanBP3 in the nucleus. Representative confocal images of middle slices of endogenous RanBP3 in double-starved NIH3T3 cells without (- P) or with PDGF (50 ng/ml) treatment for 30 min (+ P) reveals the primary localization of RanBP3 in the nucleus. n = three experiments. Scale bar =10 µm.

Supplementary Figure 8

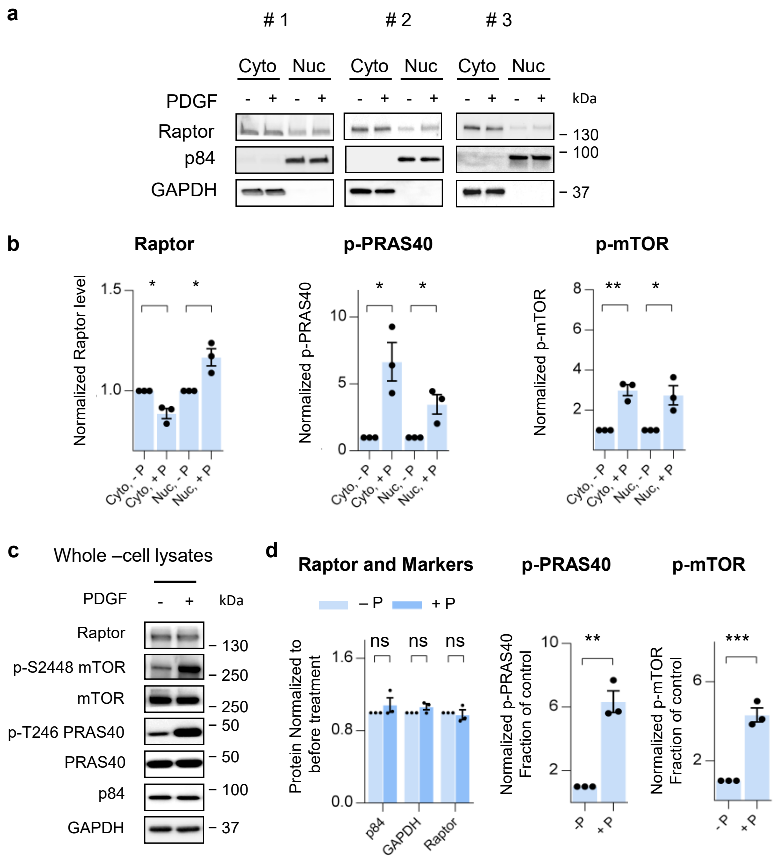

Supplementary Fig. 8 Quantification of nuclear translocation and whole cell lysates without or with PDGF stimulation in three independent experiments. a. Three independent fractionation experiments, shown by western blots of cytoplasmic and nuclear fractions from double-starved NIH3T3 cells treated without or with PDGF (50 ng/ml) for 30 min. p84 and GAPDH were used as nuclear and cytoplasmic markers, respectively. Full blots are shown in Supplementary Fig. 14. **b.** Quantification of changes in relative Raptor protein levels, phosphorylation of PRAS40 and mTOR in cytoplasmic and nuclear fractions between nontreated and treated samples. The levels of the target proteins were normalized to each respective control (GAPDH for cytoplasmic fractions and p84 for nuclear fraction), and the changes between non-treated and treated samples were compared by setting the pre-stimulation condition as 1. Data are representative of three experiments. Values are means ± s.e.m. P value was determined by unpaired two-tailed t-test. For comparison of Raptor protein levels, Cyto, -P vs. Cyto, +P, *, p=0.011; Nuc, -P vs. Nuc, +P, *, p=0.017. For comparison of p-PRAS40, Cyto, -P vs. Cyto, +P, *, p=0.017; Nuc, -P vs. Nuc, +P, *, p=0.028. For comparison of p-mTOR, Cyto, -P vs. Cyto, +P, *, p=0.002; Nuc, -P vs. Nuc, +P, *, p=0.022. **c.** Western blot analysis of the whole-cell lysates of double-starved NIH3T3 cells treated without or with PDGF (50 ng/ml) for 30 min. Full blots are shown in Supplementary Fig. 14. **d.** Quantification of relative Raptor, GAPDH, and p84 protein levels, phosphorylation of PRAS40 and mTOR in **(c)** after normalizing to the corresponding untreated conditions. Data are representative of three experiments. Values are means ± s.e.m. P value was determined by unpaired two-tailed t-test. p84, ns, not significant, p=0.018; GAPDH, ns, not significant, p=0.30; Raptor, ns, not significant, p=0.70; p-PRAS40, **, p=0.0014; p-mTOR, ***, p=0.0007.

.

Supplementary Figure 9

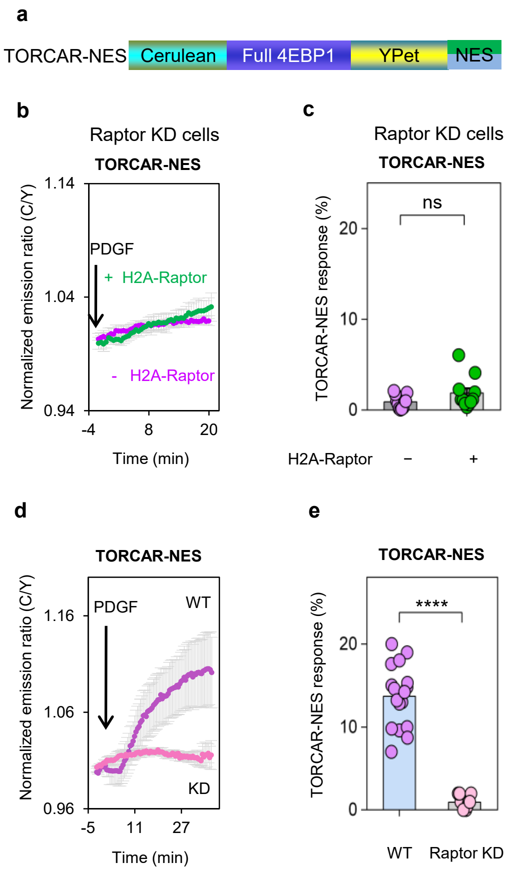

Supplementary Fig. 9 Nuclear localized Raptor does not affect cytosolic mTORC1 activity. a. Domain structure of cytosolic TORCAR (TORCAR-NES). **b.** Average time courses of normalized emission ratio (Cyan/Yellow) in double-starved Raptor knockdown NIH3T3 cells expressing cytosol targeted TORCAR (TORCAR-NES) stimulated with 50 ng/ml of PDGF without (magenta trace, n = 11 cells from 4 experiments) or with expression of H2A-Raptor (green trace, n = 11 cells from 3 experiments). Error bar represents mean ± s.e.m. **c**. Responses of TORCAR-NES in PDGF-treated double-starved Raptor knockdown NIH3T3 cells with (n = 11 cells from 3 experiments) or without H2A-Raptor expression (n = 11 cells from 4 experiments). Error bar represents mean ± s.e.m. P value was determined by unpaired two-tailed student's t-test with Welch's correction. ns, not significant, p=0.1. Source data are provided as a Source Data file. **d**. Average time courses of normalized emission ratio (Cyan/Yellow) in double-starved wildtype (WT, purple trace, n = 17 cells from 3 experiments) or Raptor knockdown (KD, light pink trace, n = 11 cells from 3 experiments) NIH3T3 cells expressing cytosol-targeted TORCAR (TORCAR-NES) stimulated with 50 ng/ml of PDGF. **e**. Responses of TORCAR-NES in PDGF-treated double-starved wildtype (n = 17 cells from 3 experiments) or Raptor knockdown NIH3T3 cells (n = 11 cells from 3 experiments). Error bar represents mean ± s.e.m. P value was determined by unpaired two-tailed student's t-test with Welch's correction.****, $p=7 \times 10^{-11}$. Source data are provided as a Source Data file.

Supplementary Figure 10

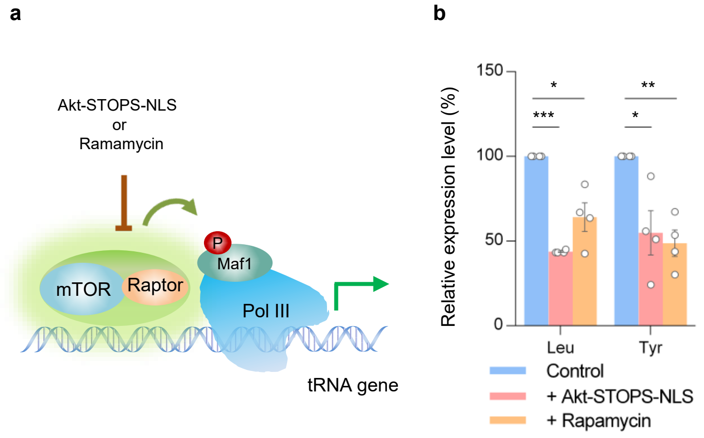

Supplementary Fig. 10 Nuclear Akt-STOPS suppresses pol III transcription. a. A model depicting phosphorylation of Maf1 by mTORC1 inactivates Maf1 as a repressor of RNA polymerase III (pol III), leading to upregulated transcription of tRNA. **b.** Akt-STOPS-NLS suppresses mTORC1-mediated Pol III transcription. Quantification of Leu tRNA and Tyr tRNA levels by real time qPCR analysis. Actin was used as normalization control. Data indicate mean values of four independent experiments and error bars refer to standard error between experiments. Statistical analyses were performed using two-way ANOVA followed by Dunnet's multiple comparisons tests. For comparison of relative expression level of Leu tRNA, Control vs. Akt-STOPS-NLS, ****, $p=3 \times 10^{-5}$; Control vs. Rapamycin, **, $p=2 \times 10^{-3}$. For comparison of relative expression level of Tyr tRNA, Control vs. Akt-STOPS-NLS, ***, $p=3 \times 10^{-4}$; Control vs. Rapamycin, ***, $p=1 \times 10^{-4}$. Source data are provided as a Source Data file.

Supplementary Figure 11

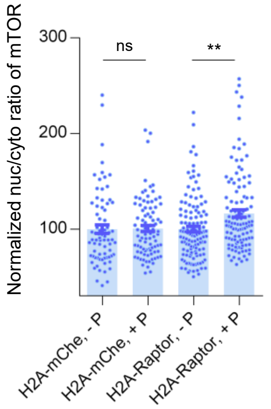

Supplementary Fig. 11 Expression of nuclear Raptor slightly increases nuclear distribution of mTOR. Quantification of mean nuclear intensity/mean cytosol intensity ratio (Nuc/Cyto ratio) of mTOR immunostaining per cell. Double-starved NIH3T3 cells expressing H2A-mCherry or H2A-Raptor without treatment (-P) or were treated with 50 ng/ml of PDGF for 30 min (+ P). n = 76, 90, 124, 117 cells. n = three experiments. All data are mean ± s.e.m. P value was determined by unpaired two-tailed student's t-test with Welch's correction. H2A-mChe, - PDGF vs. H2A-mChe, + PDGF, ns, not significant, p=0.9; H2A-Raptor, - PDGF vs. H2A-Raptor, + PDGF, **, $p=1 \times 10^{-3}$. Source data are provided as a Source Data file.

Supplementary Figure 12

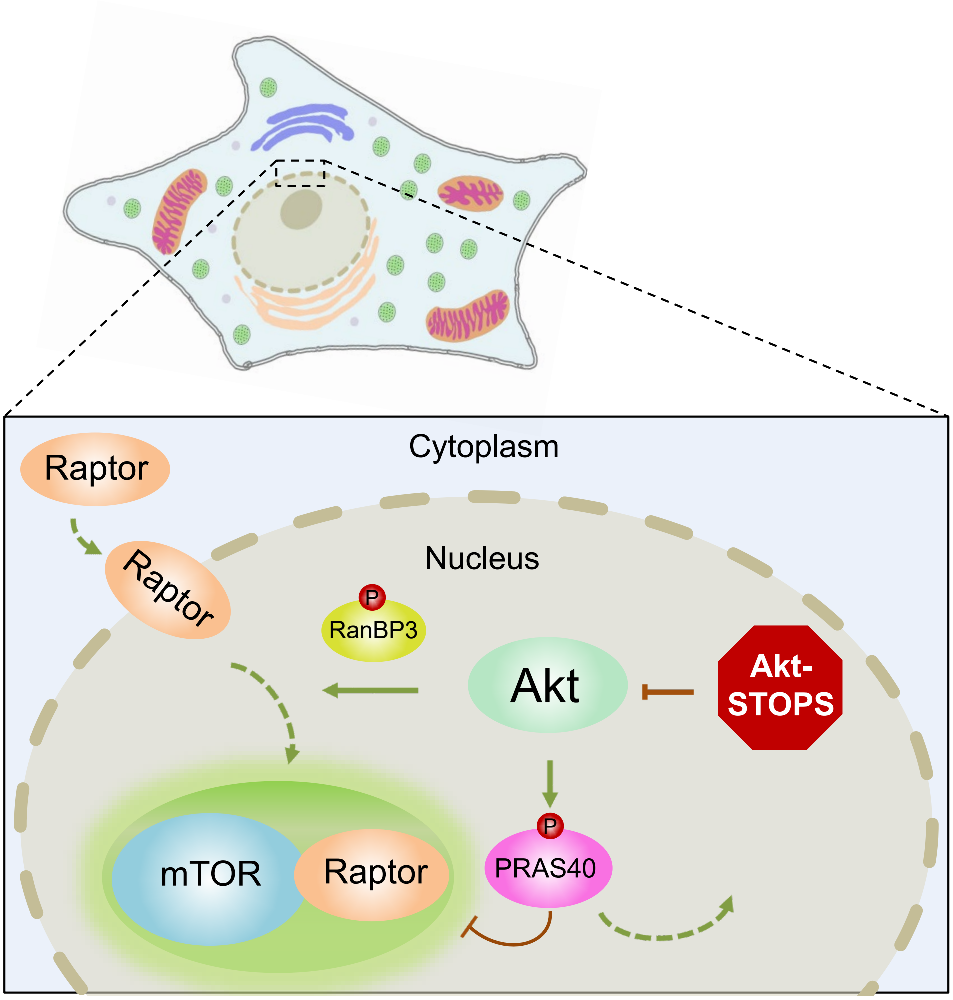

Supplementary Fig. 12 A model showing the regulation of nuclear mTORC1 by Akt. Nuclear Akt enhances the nuclear translocation of Raptor through phosphorylation of RanBP3 and relieves the negative regulation of PRAS40 on mTORC1 through phosphorylation of PRAS40. As a result, mTORC1 activity increases upon growth factor stimulation. Nuclear Akt-STOPS selectively suppresses the nuclear Akt activity, thereby reducing nuclear mTORC1 activity.

Supplementary Figure 13

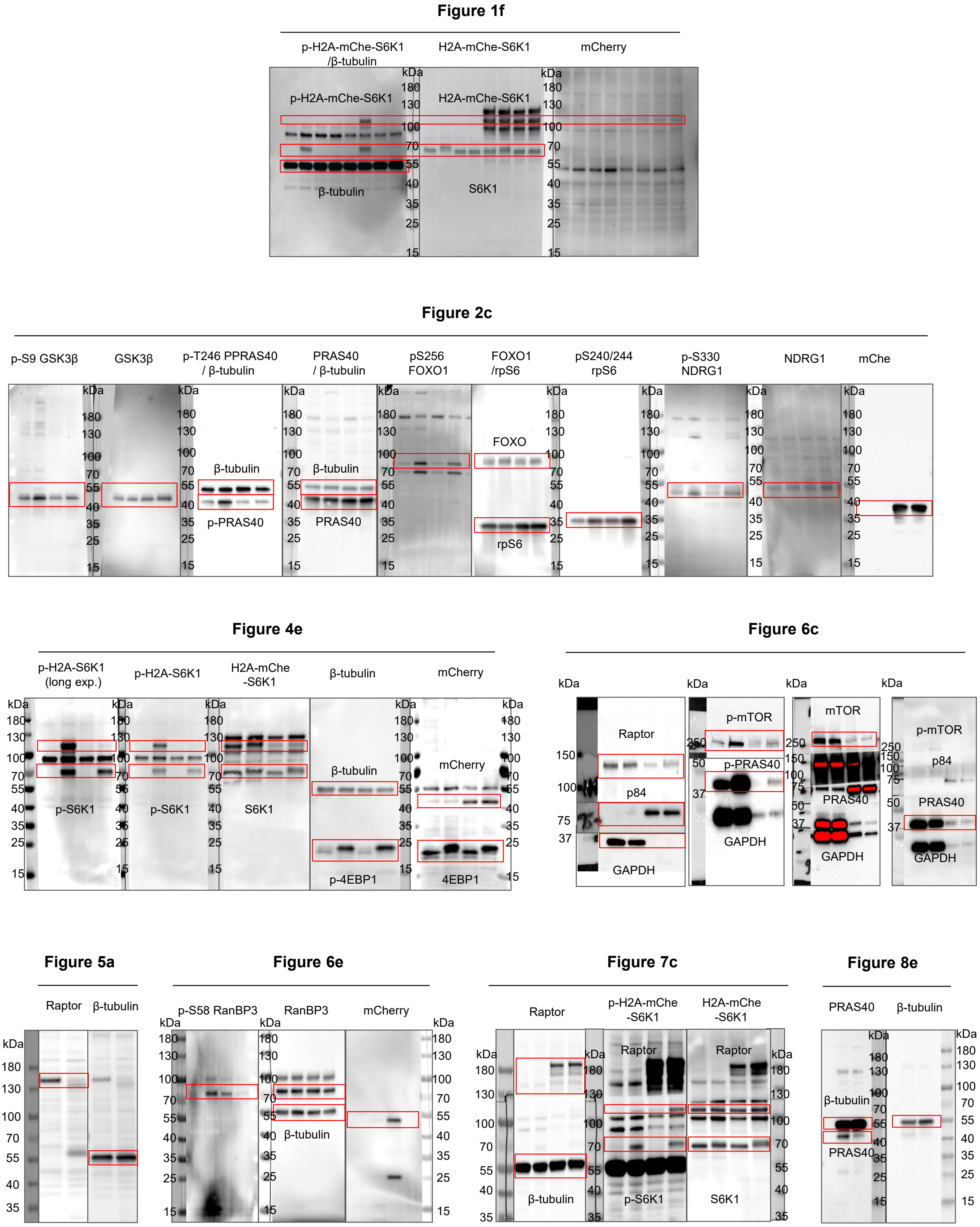

Supplementary Fig. 13 Uncropped western blots from Fig. 1-8. Uncropped images of western blots from Fig. 1, 2, 4, 5, 6,7 and 8 are shown, as well as the approximate extent of the cropped region.

Supplementary Figure 14

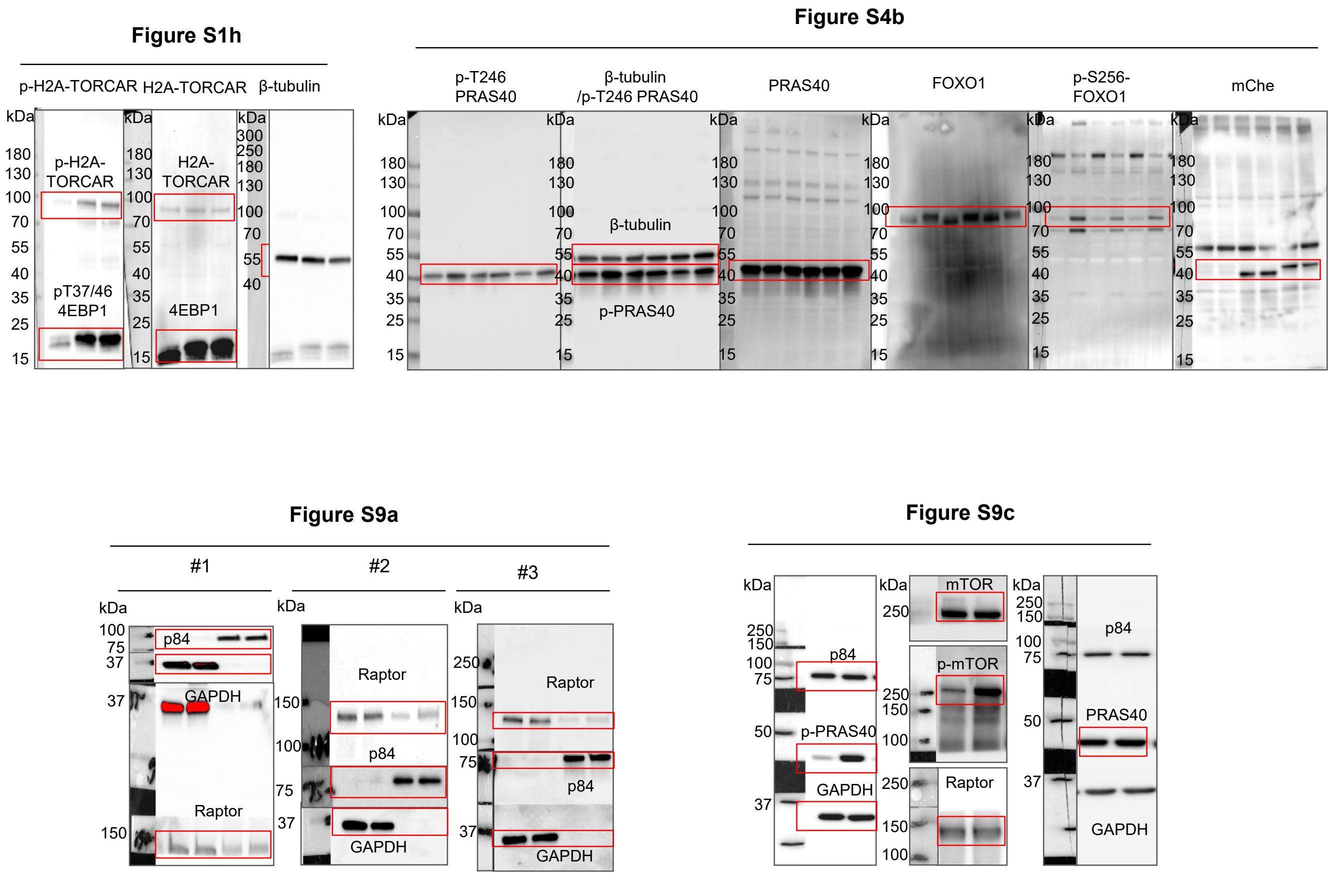

Supplementary Fig. 14 Uncropped western blots from Supplementary Figures. Uncropped images of western blots from Supplementary Fig. 1h, 4b, 8a, and 8c are shown, as well as the approximate extent of the cropped region.

Supplementary Table 1

Supplementary Table 1 Sequences of Primers.

Primer Name	Sequence 5' to 3'
SphI-AktSub-SalI-F	CATCCTCGTCCGCGCTCGTGCACCTGGCCGGACCCCAGGCCGGAGTTTGGAG
SphI-AktSub-SalI-R	TCGACTCCAAACTCCGGCCTGGGGTCCGGCCAGGTGCACGAGCGCGGACGAGGATGCATG
SacI-AktSub-EcoRI-F	CCCTCGTCCGCGCTCGTGCACCTGGCCGGACCCCAGGCCGGAGTTTG
SacI-AktSub-EcoRI-R	AATTCAAACTCCGGCCTGGGGTCCGGCCAGGTGCACGAGCGCGGACGAGGGAGCT
SacI-AktSub-stop-EcoRI-R	AATTCTTAAAACTCCGGCCTGGGGTCCGGCCAGGTGCACGAGCGCGGACGAGGGAGCT
SphI-SP-SalI-F	CATACCCCCTGCCCGAGCGAGTGGCCGCGCCGCCCGCGCGATCCATTTGGAG
SphI-SP-SalI-F	TCGACTCCAAATGGATCGCGCGGGCGGCGCGGCCACTCGCTCGGGCAGGGGGTATGCATG
SalI-SP-SacI-F	TCGACACCCCCTGCCCGAGCGAGTGGCCGCGCCGCCCGCGCGATCCATTTGGAGGTACCGG CGGCAGCGAGCT
SalI-SP-SacI-R	CGCTGCCGCCGGTACCTCCAAATGGATCGCGCGGGCGGCGCGGCCACTCGCTCGGGCAGGG GGTG
SacI-SP-EcoRI-F	CACCCCCTGCCCGAGCGAGTGGCCGCGCCGCCCGCGCGATCCATTTTAAG
SacI-SP-EcoRI-R	CACCCCCTGCCCGAGCGAGTGGCCGCGCCGCCCGCGCGATCCATTTG
SacI-SP-stop-EcoRI-R	AATTCAAATGGATCGCGCGGGCGGCGCGGCCACTCGCTCGGGCAGGGGGTGAGCT
HindIII-H2A-BamHI-F	GCGCAAGCTTGCGGCCGCCACCATGTCTGGTCGTGGCAAACAAGG
HindIII-H2A-BamHI-R	GCGCGGATCCGCTTTGCTTTTGGCTTTGTGGCTTTCGGTTTTCTTTGG
SphI-S6K1-stop-EcoRI-F	GCGCGCATGCTATGAGGCGACGAAGGAGGCGGGACGGC
SphI-S6K1-stop-EcoRI-R	GGCCAGAGCACCTGCGTATGAATCTATAGGAATTCGCGC
SphI-Raptor-stop-EcoRI-F	GCGC GCATGCATATGGAGTCCGAAATGCTGC
SphI-Raptor-stop-EcoRI-R	GCGC GAATTCCTATCTGACACGCTTCTCCAC
BamHI-mCherry-stop-EcoRI-F	GCGC GGATCCCATGGTGAGCAAGGGCGAGGAGGATAAC
BamHI-mCherry-stop-EcoRI-R	GCGCGAATTCTTA GCGCTTGTACAGCTCGTCCATGCC
BamHI-mCherry-EcoRI-R	GCGCGAATTCGCGCTTGTACAGCTCGTCCATGCC
Leu tRNA-F	GTCAGGATGGCCGAGTGGTCTAAG
Leu tRNA-R	CCACGCCTCCATTCGGAGACCAGAACACCC
Tyr tRNA-F	CCTTCGATAGCTCAGCTGGTAGAGCGGAGG
Tyr tRNA-R	CGGAATTGAACCAGCGACCTAAGGATGTCC
β-actin-F	GAGCACAGAGCCTCGCCTTT
β-actin-R	TCATCATCCATGGTGAGCTGG
