# Supplementary material for: Location-specific inhibition of Akt reveals regulation of mTORC1 activity in the nucleus
Source: Nat Commun. 2020 Nov 30;11:6088. doi: 10.1038/s41467-020-19937-w (PMC7705703; doi:10.1038/s41467-020-19937-w)
Supplement: Supplementary file 2 — Reporting Summary [file 41467_2020_19937_MOESM2_ESM.pdf]

nature research

Corresponding author(s): Jin Zhang
Last updated by author(s): Oct 17, 2020

Reporting Summary

Nature Research wishes to improve the reproducibility of the work that we publish. This form provides structure for consistency and transparency in reporting. For further information on Nature Research policies, see Authors & Referees and the Editorial Policy Checklist.

Statistics

For all statistical analyses, confirm that the following items are present in the figure legend, table legend, main text, or Methods section.

n/a	Confirmed	
☐	☒	The exact sample size (*n*) for each experimental group/condition, given as a discrete number and unit of measurement
☐	☒	A statement on whether measurements were taken from distinct samples or whether the same sample was measured repeatedly
☐	☒	The statistical test(s) used AND whether they are one- or two-sided<br>*Only common tests should be described solely by name; describe more complex techniques in the Methods section.*
☐	☒	A description of all covariates tested
☐	☒	A description of any assumptions or corrections, such as tests of normality and adjustment for multiple comparisons
☐	☒	A full description of the statistical parameters including central tendency (e.g. means) or other basic estimates (e.g. regression coefficient) AND variation (e.g. standard deviation) or associated estimates of uncertainty (e.g. confidence intervals)
☐	☒	For null hypothesis testing, the test statistic (e.g. *F*, *t*, *r*) with confidence intervals, effect sizes, degrees of freedom and *P* value noted<br>*Give P values as exact values whenever suitable.*
☒	☐	For Bayesian analysis, information on the choice of priors and Markov chain Monte Carlo settings
☒	☐	For hierarchical and complex designs, identification of the appropriate level for tests and full reporting of outcomes
☒	☐	Estimates of effect sizes (e.g. Cohen's *d*, Pearson's *r*), indicating how they were calculated

Our web collection on statistics for biologists contains articles on many of the points above.

Software and code

Policy information about availability of computer code

Data collection	Live-cell imaging data were collected using METAFLUOR 7.7. Immunofluorescence data were collected using Zen Black 2.3 SP1(Zeiss).
Data analysis	Raw imaging data were analyzed using METAFLUOR 7.7 or ImageJ 1.52s as described in the Methods. Curve fitting was performed using CFTool (3.4.1) in MatLab 8.3.0.532 (R2014A). Cell segmentation analysis and quantification were carried out using CellProfiler 3.0.0 software. Statistical analyses were performed using GraphPad Prism 6. No custom code was used.

For manuscripts utilizing custom algorithms or software that are central to the research but not yet described in published literature, software must be made available to editors/reviewers. We strongly encourage code deposition in a community repository (e.g. GitHub). See the Nature Research guidelines for submitting code & software for further information.

Data

Policy information about availability of data

All manuscripts must include a data availability statement. This statement should provide the following information, where applicable:

- Accession codes, unique identifiers, or web links for publicly available datasets
- A list of figures that have associated raw data
- A description of any restrictions on data availability

The authors declare that all the data supporting the findings of this study are available within the paper and its supplementary information files or from the corresponding author upon reasonable request. Source data are provided with this paper.

Field-specific reporting

Please select the one below that is the best fit for your research. If you are not sure, read the appropriate sections before making your selection.

☒ Life sciences ☐ Behavioural & social sciences ☐ Ecological, evolutionary & environmental sciences

For a reference copy of the document with all sections, see nature.com/documents/nr-reporting-summary-flat.pdf

Life sciences study design

All studies must disclose on these points even when the disclosure is negative.

Sample size	No sample-size calculations were performed. Sample sizes were similar to those generally employed in the field. For single cell imaging please refer to Mehta, Zhang, and Roth et al. 2018 Nat. Cell. Biol. and Keyes et al. 2020 eLife.
Data exclusions	Figure 2b,d,e; 3a-d; 5a-b; Supplementary Figure 3d, 4a, 5b-d, 6b-c, 10b-e: Cells that did not express all of a given set of constructs (for multi-color imaging experiments) were excluded from analysis. All exclusion criteria were pre-determined.
Replication	Unless otherwise noted, all experiments were repeated at least three times. All replication attempts were successful.
Randomization	Cells were randomly seeded into experimental groups after resuspension.
Blinding	Blinding was not performed, it was not applicable to our study as it is not worked with participants. Objective quantitative data was generated and analyzed in all experiments, and no subjective classifications were performed.

Reporting for specific materials, systems and methods

We require information from authors about some types of materials, experimental systems and methods used in many studies. Here, indicate whether each material, system or method listed is relevant to your study. If you are not sure if a list item applies to your research, read the appropriate section before selecting a response.

Materials & experimental systems

n/a	Involved in the study	
☐	☒	Antibodies
☐	☒	Eukaryotic cell lines
☒	☐	Palaeontology
☒	☐	Animals and other organisms
☒	☐	Human research participants
☒	☐	Clinical data

Methods

n/a	Involved in the study	
☒	☐	ChIP-seq
☒	☐	Flow cytometry
☒	☐	MRI-based neuroimaging

Antibodies

Antibodies used

Primary antibodies used for immunoblotting:
1. From Cell Signaling Technology: p-4EBP1 (T37/46) (#2855), 4EBP1 (#9452), p-S6K1 (T389) (#9205), S6K1 (#9202), p-GSK3β (S9) (#9322), p-PRAS40 (T246) (#2997), PRAS40 (#2691), p-NDRG1 (S330) (#3506), NDRG1 (#5196), p-rpS6 (S240/244) (#5364), rpS6 (#2317), p-FoxO1 (S256) (#9461), FoxO1 (#2880), p-RanBP3 (S58) (#9380) and tubulin (#2146) antibodies
2. From BD Bioscience: GSK3β (#610201) antibody
3. From Invitrogen: RanBP3 (700076) antibody
4. From ProteinTech: Raptor antibody (20984-1-AP)
5. From GenScript: RFP antibody (A00682-100).

Secondary antibodies used for immunoblotting:
The horseradish peroxidase-labeled goat anti-rabbit (PI31460) or anti-mouse (PI31430) were purchased from Pierce.

Antibodies used for the PLA assay:
anti-mTOR(66888-1-Ig) and anti-Raptor (20984-1-AP) from ProteinTech,
anti-mTOR(#2983) and normal rabbit IgG (#2792) from Cell Signaling.

Antibodies used for immunofluorescence:
Raptor (20984-1-AP, Proteintech, 1:100),
mTOR (#2983, Cell Signaling, 1:100),
p-PRAS (T246) (#2997, Cell Signaling, 1:100),
PRAS (ThermoFisher, AHO1031, 1:100),
RanBP3 (Invitrogen, 700076, 1:100),
anti-rabbit Alexa Fluor 488 (A11006) and

	anti-mouse Alexa Fluor 568(A11004) from Life Technologies/Molecular Probes.
Validation	All the antibodies used in the study are common, well-established commercial antibodies. Validation data are shown for each antibody by the manufacturers. Rabbit anti-Raptor antibody (20984-1-AP) was validated by vendor ProteinTech, and the validation was verified using KD cells in our study.

Eukaryotic cell lines

Policy information about cell lines

Cell line source(s)	HEK293T, NIH3T3, and differentiated 3T3L1 adipocytes were used in this study. All cell lines were obtained from ATCC.
Authentication	Cell line identities were verified via STR analysis by the commercial source and then maintained separately and isolated from one another to avoid cross-contamination.
Mycoplasma contamination	All cell lines were determined to be free of mycoplasma contamination based on weekly DNA staining.
Commonly misidentified lines (See ICLAC register)	There are no commonly misidentified cell lines.
